# Spatio-temporal trends and socio-environmental determinants of suicides in England (2002–2022): an ecological population-based study

**DOI:** 10.1016/j.lanepe.2025.101386

**Published:** 2025-08-14

**Authors:** Connor Gascoigne, Annie Jeffery, Ioannis Rotous, Xuewen Yu, Sara Geneletti, Bethan Davies, Gianluca Baio, James B. Kirkbride, Alexandra Pitman, Marta Blangiardo

**Affiliations:** a MRC Centre for Environment and Health, Department of Epidemiology and Biostatistics, School of Public Health, Imperial College London, London, UK; b Division of Psychiatry, University College London, London, UK; c Department of Statistical Science, University College London, London, UK; d Department of Statistics, London School of Economics and Political Science, London, UK; e Small Area Health Statistics Unit (SAHSU), Department of Epidemiology and Biostatistics, School of Public Health, Imperial College London, London, UK; f North London NHS Foundation Trust, London, UK

**Keywords:** Suicide, Mental health, Public health, Spatio-temporal analysis, Spatial inequalities, Socio-environmental determinants, Ecological study

## Abstract

**Background:**

Over the last two decades of suicide prevention strategy implementation, suicide rates in England have shown a fluctuating pattern, declining from the early 2000s (10.3 deaths per 100,000 in 2002) until around 2010 (9.0 deaths per 100,000 in 2007), then gradually increasing (10.7 deaths per 100,000 in 2022). It remains unclear whether the pattern varies by local area, the influence of the socio-environmental factors or a combination of both. Our aim was to evaluate spatio-temporal trends of suicides in England from 2002 to 2022 whilst examining the role of socio-environmental characteristics.

**Methods:**

In this ecological study, we analysed Office for National Statistics data on deaths by suicide, exploring spatial and temporal patterns in England (2002–2022). Using a Hurdle Poisson model fit within a Bayesian hierarchical framework, we assessed the effects of local area level deprivation, ethnic density, population density, light pollution, railway and road network densities and greenspace composition on suicide risk.

**Findings:**

From 2002 to 2022, suicide risk across England showed no substantial change overall (−4.26%; 95% Credible Interval (CrI): −8.95%, 0.72%). The difference between the regions with the lowest (London) and highest (North East) risk was 39.2% (95% CrI: 34.1%, 44.3%). We found that for one standard deviation change in each covariate, suicide risk increased with deprivation (20.06%; 95% CrI: 18.48%, 21.65%), railway network density (1.37%; 95% CrI: 0.32%, 2.46%), and road network density (5.16%; 95% CrI: 3.12%, 7.46%) while risk decreased with ethnic density (−7.47%; 95% CrI: −8.91%, −6.00%), population density (−5.42%; 95% CrI: −7.34%, −3.25%), light pollution (−4.20%; 95% CrI: −5.71%, −2.72%), and greenspace composition (−6.43%; 95% CrI: −7.94%, −4.99%).

**Interpretation:**

We did not find evidence to support a decline in suicide rates in England over the last 20 years and our findings highlight the community profiles, characterised by greater deprivation, isolation, and access to road/rail networks, where suicide risk was highest. This should help focus future research to understand these as drivers of suicide risk, leading to the development of effective area-level interventions and targeted investment in those approaches where most needed.

**Funding:**

10.13039/100010269Wellcome Trust, UKHSA, 10.13039/501100000265MRC, 10.13039/501100000272NIHR through its 10.13039/100018336HPRU, 10.13039/501100023699HDRUK, 10.13039/501100012621NIHR University College Hospital London (UCLH) Biomedical Research Centre (BRC).

**Research in context**

**Evidence before this study**

Globally, there are over 720,000 suicide deaths annually. It is important to understand the role of geographical, social, and environmental factors, to identify potentially vulnerable communities and inform future prevention strategies. We searched PubMed for relevant articles using the following key words: “spatial” and “temporal” or “spatio-temporal” combined with “ecological” or “longitudinal” combined with “suicide”. The 26 articles returned provided evidence of spatial clustering, temporal trends, and associations between some socioeconomic factors and suicides. As suicides are a relatively rare outcome, there was a tendency for the scientific papers identified to consider large geographical areas and/or time periods to limit data sparsity. Consequently, they had limited scope to investigate local socio-environmental factors and short follow up periods for temporal analyses. This highlights the need for population-based studies at high spatio-temporal resolution with the capacity to investigate local temporal trends in suicides whilst also investigating the influence of local socio-environmental characteristics.

**Added value of this study**

Our study is the first to explore high-resolution spatio-temporal trends in suicides whilst exploring the influence of local socio-environmental factors. We specified a spatio-temporal model for suicide counts in England from 2002 to 2022 using fine-grained area-level mortality data from the UK Office for National Statistics (for 6791 areas in England). We considered the influence of specific socio-environmental factors at the small area-level: deprivation, ethnic density, population density, light pollution, railway and road network densities, and greenspace composition.

Comparing suicide risk in England across the study period, we found no evidence that suicide risk varied across England in the years 2002–2022. However, there was considerable year-on-year variation as well as variation across local areas. We demonstrated that suicide rates were higher in areas with high levels of deprivation, and railway and road network densities and those suicides were lower in areas with high levels of ethnic density, population density, light pollution and greenspace.

**Implications of all the available evidence**

The lack of a substantial change in population suicide risk over the study period would suggest a need to improve the evidence underpinning suicide prevention strategy in England. In particular, our study highlights the role of community-level characteristics on suicide risks, and we suggest that suicide prevention efforts should focus more on understanding the influence of local socio-environmental factors, targeting these to reduce geographical, social, and environmental disparities.

## Introduction

An average of 6311 (11.0 per 100,000) suicide deaths occur annually in the United Kingdom (UK), with yearly suicide rates across all nations (England, Northern Ireland, Scotland, and Wales) fluctuating around similar values for the last decade.^1^ Meanwhile, the European Union (EU) has successfully reduced its suicide rate from 12.4 to 10.2 deaths per 100,000.^2^ The UK is not just lagging; it has a critical opportunity, and an urgent responsibility, to do better. Each suicide death is estimated to cost the UK economy £1.46 million^3^ and has a substantial emotional impact on members of the community, estimated at between 60^4^ and 135^5^ people. The evolving nature of suicide epidemiology necessitates careful and continuous surveillance to ensure that prevention activities are targeted appropriately. For this reason, a near real-time suspected suicide surveillance (nRTSSS) system was launched in England in 2023.^6^ Whilst individual-level modifiable suicide risk factors such as untreated psychiatric illness or chronic pain are well-recognised,^7^ less is known about area-level risk factors for suicide and how they vary over time. A better understanding of these factors would help suicide prevention interventions at the regional level, where public health agencies have responsibility.

Despite the high number of deaths by suicide annually, they remain relatively rare events from a statistical perspective, creating a challenge for policymakers in understanding subnational spatial and temporal trends. This issue of data sparsity in research is often addressed by lowering spatial and/or temporal resolution to reduce zeros but sacrificing granular analysis of the potential socio-environmental factors of spatio-temporal patterns in suicide. For example, Nazari et al.^8^ analysed yearly data in Iran at regional level (31 regions), whilst Balint et al.^9^ considered a high spatial resolution in Hungary (175 micro regions) at low temporal resolution (10-year period).

Some studies have achieved analyses at high spatio-temporal resolutions; for instance, Helbich et al.^10^ and Kandula et al.,^11^ considered yearly suicide rates for 402 districts in Germany and 3142 counties in the USA, respectively. They both included socio-economic factors and found high spatial heterogeneity in suicide risk. However, the risk factors they considered were limited and neither investigated the role of environmental factors such as greenspace or noise.

In England and Wales both Middleton et al.^12^ and Congdon^13^ considered high spatial resolutions (9265 wards in England and Wales and 3242 wards in the East and South East of England, respectively). Both studies found spatial variation nationally [described as a ‘bullseye’ pattern of suicides in cities where suicide rates were highest in the inner-city and lowest on the outer city^12^] and when considering the South East of England only,^13^ respectively. However, neither included a temporal dimension, and both sets of authors stated the need for more in-depth analysis in geographical variations in suicide. Another study by Gunnell et al.^14^ explored changes in the spatial pattern of suicide rates in young men in England using three five-year periods (1981–1985, 1991–1995, and 2001–2005) and 1113 geographical areas.

In terms of socio-environmental factors, both Middleton et al.^12^ and Congdon^13^ identify social deprivation as a risk factor and urbanicity as a protective factor. Studies outside of England have risk factors of suicide to be traffic and rail exposure^15^^,^^16^ whilst population density,^10^^,^^11^ and greenspace^17^^,^^18^ are considered protective factors.

No study in England has yet provided a comprehensive picture of high spatio-temporal suicide trends over a substantial time period, which would facilitate the investigation of local area level socio-environmental factors that may drive suicide trends. Such work is required given it is closely aligned with the recommendations in the UK Government's latest suicide prevention strategy for England.^19^ Furthermore, insights provided will be critical to ensure that England sees reductions in suicide that in line with those seen in other European countries.^6^

In this paper we present a high-resolution analysis of spatio-temporal trends of suicides in England, at the same time examining the role of specific socio-environmental local areal level characteristics. Our study covers 20-years (2002–2022), which enabled us to focus on long-term trends during a period that has also witnessed rapid increases in the incidence and prevalence of many mental health conditions. At the same time, we used the highest spatial resolution ever considered in the context of suicides in England which allowed us to explore the effect of socio-environmental factors at the local area level. Additionally, we were able to identify area profiles most at risk based on combinations of empirically identified local area level risk factors, which can inform population level suicide prevention strategies. We hypothesise that in England, we will see large regional and sub-regional differences in suicide risk driven by rurality, isolation and deprivation.

## Methods

We examined spatio-temporal trends in suicide risks in England from 2002 to 2022. We considered yearly number of events in each of 6791 Middle layer Super Output Areas (MSOA) of England defined in by the 2011 census. Each MSOA is an administrative level comprising of 5000–15,000 individuals.^20^ Data were stratified by age ([15, 25), [25, 35), [35, 45), [45, 55), [55, 65), [65, 75), [75, 85), 85+, and sex (male/female).

### Outcome

Yearly suicide counts, by age, sex and MSOA of residence were obtained from the Office for National Statistics (ONS) mortality database held by the Small Area Health Statistic Unit (SAHSU) at Imperial College, London. We defined suicides using the International Classification of Diseases version 10 (ICD-10) codes X60–X84 (for intentional self-harm). Additionally, we included the codes Y10–Y34 (excluding Y33.9), Y87.0, and Y87.2 (for events of undetermined intent) as consistent with several other English suicide epidemiology studies.^21^, ^22^, ^23^ To estimate standardised mortality ratios, we also retrieved yearly population totals from the same database, stratified by the same MSOA-age-sex groups, used to calculated expected counts of suicides (see Supplementary Material).

### Socio-environmental factors

To investigate key socio-environmental factors associated with suicide risk, we considered the following seven local area level factors at MSOA level as previously considered in scientific papers. We included measures of deprivation,^12^^,^^13^ ethnic density,^24^, ^25^, ^26^ population density,^10^^,^^11^ light pollution,^27^ railway network density,^15^^,^^16^ road network density,^15^^,^^16^ and greenspace.^17^^,^^18^ All the socio-environmental factors were standardised and included in the model as continuous terms. Further details on each can be found in the Supplementary Material.

### Statistical analysis

To estimate spatio-temporal trends in suicide risk and the associations with socio-environmental factors, we used a Bayesian spatio-temporal regression model. This approach naturally overcomes issues related to data sparsity, as it assumes a hierarchical structure on the spatial and temporal domains.^28^

First, we performed an age-sex indirect standardisation to obtain adjusted expected counts of suicides for each MSOA-year combination in the study period, using the whole study region population (England, 2002–2022) as reference. To specifically account for the excess number of zeros in the data (which arise due to the high spatio-temporal resolution), we modelled suicide counts with a Hurdle Poisson (HP) model.^29^ The HP model is a two-component model where the event of a suicide (zeros/ones) and number of suicides (non-zero counts) arise from two separate data-generation mechanisms but are modelled together. We modelled the event of suicide using a binomial distribution and the number of suicides using a zero-truncated Poisson distribution and use the adjusted expected counts as offset.

We included the seven individual socio-environmental factors in the model through a regression on the parameters of the Binomial and Poisson distribution. We also modelled the residual variability in suicides through spatial, temporal, and spatio-temporal random effects. Each of these random effects were shared across the two components of the HP model. We reported the median Relative Risk (RR), which estimates the risk of suicides for each temporal unit, compared with the entire study period, or for each spatial unit compared with the average area characterised by the same population structure in terms of age and sex. Alongside the RRs, we reported estimates of the uncertainty as the 95% Credible Interval (CrI). Additionally for spatial RRs, we reported the probability the RR exceeded one, P(RR > 1) which we categorised into low, medium, and high evidence of an excess risk, as represented by [0%, 20%], (20%, 80%], and (80%, 100%], respectively.^30^

To explore the role of each socio-environmental factor on the risk of suicides, we reported the percentage change in risk of suicide for one standard deviation increment for each socio-environmental factor (adjusted for average effect of the six other socio-environmental factors as well as space and time), with the associated 95% CrI. To explore how influential the socio-environmental factors and the spatial, temporal and spatio-temporal random effects are, we reported how much of the variability in the estimated RR were explained by each. There are no guidelines on what a high or low amount of variability would be; however, this has been done previously in the context of modelling excess mortality.^31^ To evaluate which combination of socio-environmental factors had the highest impact on suicide risk, we ranked the percentiles of MSOA-year RR and reported the average socio-environmental score for each percentile of RR.

Analysis was performed in R using r-inla.^32^ A complete specification of the model can be found in Section 2 of the Supplementary Material and the full code for the analysis is provided on the GitHub https://github.com/connorgascoigne/englishSuicides.

### Ethic approval

Imperial Colleges Small Area Health Statistic Unit (SAHSU) holds approvals both from the London - South East Research Ethics Committee (22/LO/0256) and from the Health Research Authority Confidentiality Advisory Group (20/CAG/0028).

### Role of the funding source

The funders played no part in the design, data collection and analysis, or interpretation and writing up of the study.

## Results

### National and regional trends

**Fig. 1:**
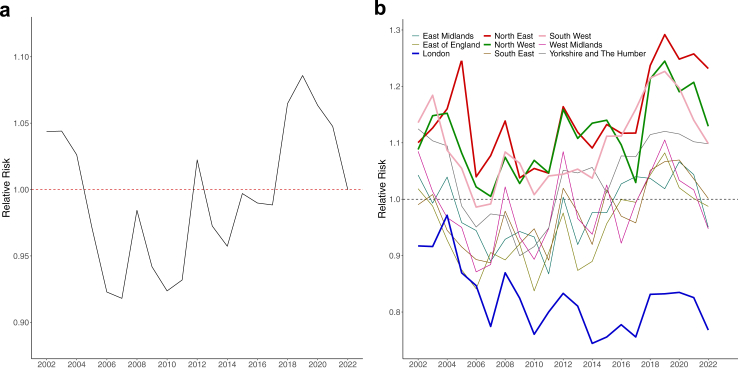
**Main:** Relative Risk over the study period nationally (left) and regionally (right). **Panel a:** National. **Panel b:** Regional.

Across the study period, from 2002 to 2022, suicide risk did not show evidence of a substantial change. The RR in 2002 and 2022 was estimated at 1.04 (95% CrI: 1.01, 1.08) and 1.00 (95% CrI: 0.97, 1.04), respectively, with an overall change in risk of −4.26% (95% CrI: −8.95%, 0.72%). At the same time, we observed large year-on-year fluctuations in the RR from 2002 to 2022 (see Fig. 1, panel (a)). Suicide risk fell sharply after 2002 and remained low over the next decade, with the lowest value of 0.92 (95% CrI: 0.88, 0.95) estimated for 2007. After 2012, there was an initial decrease in suicide risk for 2013 and 2014 followed by a steady climb in suicide risk until 2019, for which we estimated a peak of 1.09 (95% CrI: 1.05, 1.12). In the final years of the study period, the suicide risk reduced and ended being as expected.

Spatially, the North East (red) and the North West (green) indicated a higher than average suicide mortality for all years in the study period, with mean RR above 1. Additionally, the South West (pink) had consistently higher RRs in all years but 2006 (0.99; 95% CrI: 0.92, 1.06) and 2007 (0.99; 95% CrI: 0.92, 1.06) where the RR was below one. In contrast, London (blue) was the only region where the RR for all years was below one, indicating that suicide mortality was consistently lower than the national average. Over the entire study period, the suicide risk in London was 39.2% (95% CrI: 34.1%, 44.3%) lower than in the North East of England, the region characterised by the highest risk (see Fig. 1, panel (b)).

### Spatio-temporal trends

**Fig. 2:**
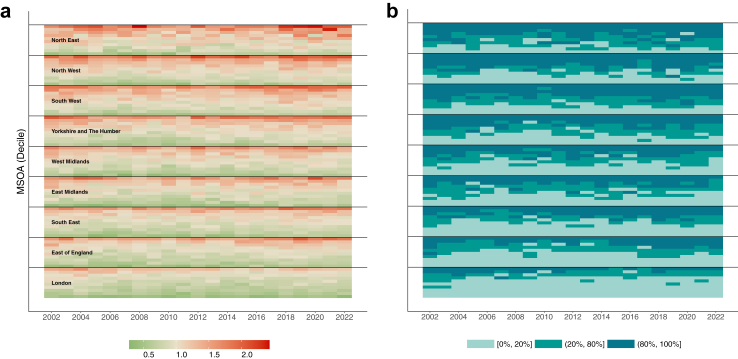
**Main:** Relative Risk (RR) and their exceedance (of one) for MSOA (decile)-year combinations. The MSOAs deciles are ordered (bottom-to-top on the $y$-axis) within region from the lowest average RR to highest average RR across the study period. Furthermore, the regions are ordered (bottom-to-top on the $y$-axis) from lowest-to-highest average RR across the entire study period. The left-hand plot shows the posterior median of the RR and the right-hand plot shows (as a percentage) how often the RR is greater than one. **Panel a:** RR. **Panel b:** P(RR > 1).

Fig. 2 shows the spatio-temporal trends for the RRs for the 6791 MSOAs grouped into deciles within their regions. The deciles are calculated based on overall RR. We presented both the RR (left) and the probability that the RR was greater than one (P(RR > 1); right). Across regions, we observed different degrees of variability for the year-on-year RR. The yearly RR for suicide in the London MSOAs showed a similar pattern across the entire study period, while the yearly RR for MSOAs in both the North East and North West were more variable. In general, there was greater within-region variability of the RR for suicide in the latter years (i.e., from 2012 onwards) of the period of interest. The highest RRs occurred at the start of the study period (in the first five years, 2002–2007) and at the end of the study period (in the last four years, 2018–2022).

As a region, the yearly RR for London was always below one. However, sub-regionally in London, this was not the case for many of the MSOAs in the top two deciles, which presented a RR above one. Inspecting the exceedance probability confirmed this variability, with values above 80% for most MSOAs in the top decile, suggesting strong evidence of increased risk of suicide for these areas. Conversely, while the majority of MSOAs in the North East and North West were characterised by a RR above one, we noted that all regions in the bottom decile of the North East and bottom two deciles of the North West had the lowest exceedance [0%, 20%], suggesting a lack of evidence to support increased risk of suicide.

### Socio-environmental factors influence

**Fig. 3:**
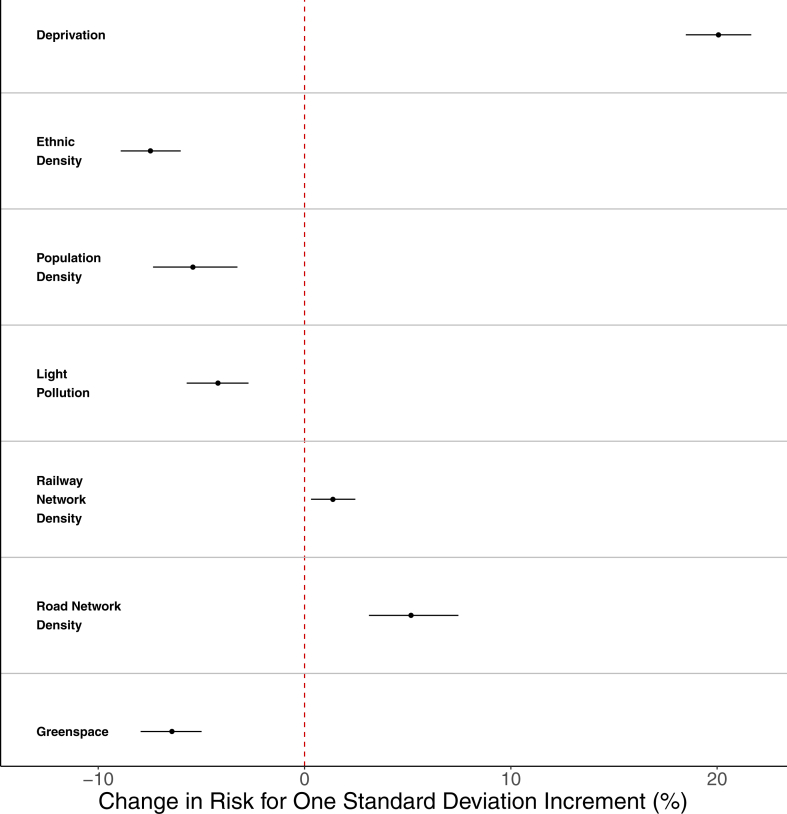
Percent change in the risk of suicide for one standard deviation increment in each of the socio-environmental factors (adjusted model).

We presented values for the percentage change in risk of suicide for one standard deviation increment for each of the seven socio-environmental factors in the fully adjusted model (Fig. 3). Deprivation showed the highest effect, with a risk change of 20.06% (95% CrI: 18.48%, 21.65%). Road and railway network densities were also characterised by a positive association with suicide, but the estimates were much smaller (1.37%; 95% CrI: 0.32%, 2.46% for railway, 5.16%; 95% CrI: 3.12%, 7.46% for road). Ethnic density showed the highest inverse relationship with suicides, with areas that were more ethnically diverse being less at risk of suicide (−7.47%; 95% CrI: −8.91%, −6.00%). Similarly, the risk of suicides was lower in areas with larger proportions of green space (−6.43%; 95% CrI: −7.94%, −4.99%), higher population density (−5.42%; 95% CrI: −7.34%, −3.25%), and higher levels of light pollution (−4.2%; 95% CrI: −5.71%, −2.72%).

We present the results separately for the Binomial and Poisson regression model in Table 3 of the Supplementary Material, but stress that the interpretation and the effect sizes are consistent with those reported here.

The seven socio-environmental factors included in the model explained 38.95% (95% CrI: 34.54%, 43.40%) of the total spatio-temporal variability observed in suicide risks, while a further 55.13% (95% CrI: 49.86%, 60.10%) was explained by residual spatial variation at the local area level (the spatial term). Finally, the temporal and spatio-temporal terms explained 5.51% (95% CrI: 4.58%, 6.61%) and 11.67% (95% CrI: 5.53%, 18.74%) of the variation, respectively.

### Full socio-environmental profiles

**Fig. 4:**
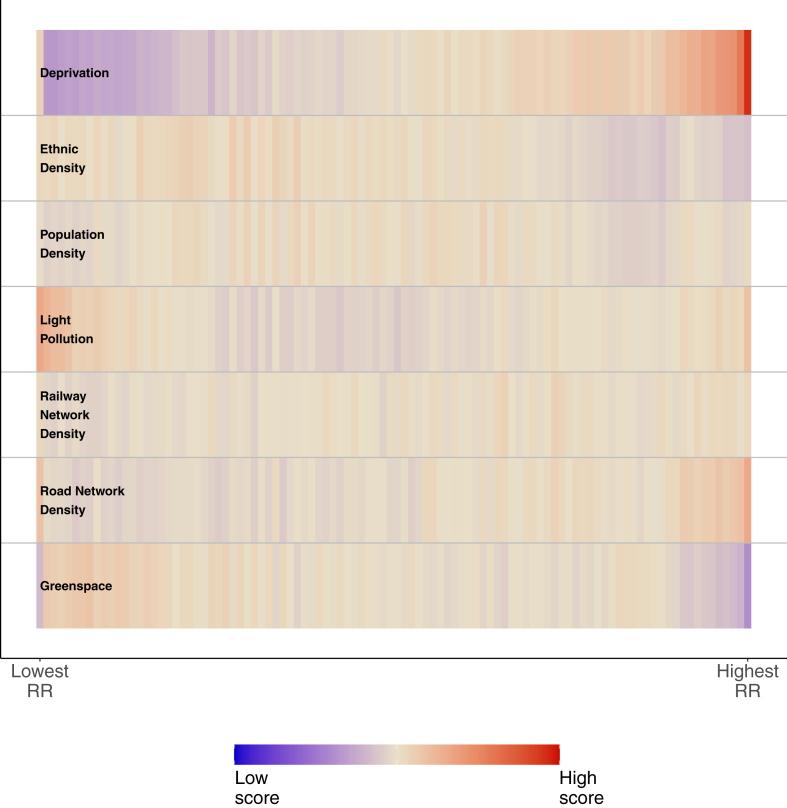
Average socio-environmental score for MSOA percentiles.

We presented the socio-environmental profiles for the MSOA percentiles ranked by the values of their RR (Fig. 4) to highlight which combination of local characteristics were associated with high/low suicide risks.

Focussing on the areas with the highest suicide risk (right-hand side of Fig. 4), we observed these were characterised consistently by high deprivation and road network density scores alongside a low score for greenspace composition. However, for ethnic diversity, population density and light pollution, there was no clear high/low score associated with higher suicide risk. The areas with the lowest suicide risk (left-hand side of Fig. 4), were characterised consistently by lowest scores of deprivations alongside the highest levels of light pollution and greenspace. Again, patterns for the remaining factors were less clear.

## Discussion

Overall, we found no substantial change in suicide risk over this period, but this masked yearly temporal variation. Suicide risk fell from 2002 and then remained relatively (with occasional spikes, i.e., 2012) low until gradually increase from 2014 (peaking in 2019) to the end of the study period. Our findings are broadly in line with official statistics from the ONS, which reported for England and Wales 10.3 registered suicides per 100,000 people in 2002 and 10.7 registered suicides per 100,000 people in 2022, with the years with the lowest and highest reported suicides per 100,000 being 2007 (9.0 per 100,000) and 2019 (11.0 per 100,000), respectively.^21^ Differences between our results and those from the ONS are likely due to methodological approach, combining Wales and England suicides, and registration delay.

We highlighted substantial geographical disparities in suicide risk across England, with strong evidence of sustained elevation in risk in the North West, North East, and South West of England, and of consistently low risk in London. We found that in most areas, suicide risks were positively associated with socioeconomic deprivation, railway network density, and road network density, but negatively associated with the proportion of minoritised ethnic groups, population density, light pollution, and greenspace composition.

Among the factors we found to increase the risk of suicides, deprivation has been previously reported as a risk factor in England^12^, ^13^, ^14^ and other high-income countries.^10^^,^^11^ The deprived regions with the highest estimated risks in the North East, North West, and South West of England are historically some of England's poorest communities; here, individuals can face a range of socioeconomic challenges, including social isolation, and legacies of deindustrialisation that create intergenerational exposure to adverse social factors of mental health.^33^ Our regional results were consistent with those from official statistics, which find that North East England and London report highest and lowest suicide rates, respectively.^21^ A recent Swiss study found both railway and road were positively associated with suicide^16^ whilst an English study found positive associations between individual level exposure to road traffic noise pollution and mental disorders.^15^ Different mechanisms may account for any causal association between road and railway network density and increased risk of suicide, including either greater exposure to noise or via greater access to means of suicide methods.

We found ethnic density, population density, light pollution, and greenspace were associated with lower rates of suicide. A previous study in Holland found high suicides rates in areas with a higher proportion of native Dutch compared to non-native Dutch residence.^34^ Research on the individual (person) level association between ethnicity and mortality (including suicide, specifically) in England is grossly lacking, arising because ethnicity has not historically been recorded on death certificates, creating an epistemic inequality in our understanding of ethnic disparities in mortality, including suicide epidemiology.^35^ Nonetheless, data on this issue are beginning to emerge, with three recent individual level studies in the UK reporting that suicide was more common amongst individuals who identified as White or as Mixed ethnicity compared with other ethnic groups.^24^, ^25^, ^26^ Possible explanations for lower suicide rates among minoritized ethnic groups include stronger religious and community support^36^ or misclassification, as an English study found lower suicides rates in these groups even though they had a larger number of risk factors indicating suicides were more likely to be recorded under non-suicide verdicts.^25^

The protective effects of population density, which aligns with findings from Germany^10^ and the USA,^11^ suggest the risk of suicide was higher in rural communities. The protective effect of local area level night-time light was a proxy for urbanicity (rural areas are darker at night).^37^ The results of both support the idea that rural communities (dark and sparsely populated) are areas of higher risk, potentially as people experience more isolation and loneliness.^12^^,^^14^ This emphasises the need to consider social isolation and/or greater access to means of suicide in rural communities as two potential points of intervention. Lastly, our findings on greenspace as a protective factor align with studies from the Netherlands^17^ and Belgium.^18^

Our socio-environmental profile analysis pointed towards lower suicide risk in local areas which are characterised being more affluent, having a more night-time light, and more greenspace. In contrast, the highest suicide risks were found in local areas characterised by high deprivation, road network density, and little greenspace. Both point towards cities (higher night-time light and road density, respectively) with lower risk on the outskirts (more greenspace and more affluent), than in the inner city (less greenspace and more deprived). This was in keeping with the ‘bullseye’ pattern of suicide (lower-to-higher risk moving from outer-to-inner city) present in cities.^14^

To our knowledge, this was the first study to explore spatio-temporal trends in suicide at a high spatio-temporal resolution over a long study period in England. Previous studies exploring spatio-temporal trends in English suicide trends used lower spatial^38^ or temporal resolutions^12^, ^13^, ^14^ to avoid handling the considerable number of zero values arising when considering a rare outcome. Consequently, we were able to explore an increased number of socio-environmental factors simultaneously in comparison with previous studies. Even when including these socio-environmental factors, our results highlighted the degree of unmeasured spatial confounding at the local area level, which still explained the majority of variability seen in suicide risk. Hence, we stress the importance of including a spatial random effect to capture the unmeasured local area level confounding, to minimise the effect of residual confounding on the covariates effect estimates. Additionally, identification of the spatial factors represented in the spatial residuals are an important research priority.

Our use of a Hurdle-Poisson model accounted for the high number of zero values present in the dataset and included random effects to account for unmeasured confounding in space and time. Our use of a Bayesian approach naturally allowed us to report the full uncertainty on the model estimates, for instance exploring the profiles of higher- and lower-than-expected risk in suicide mortality. The approach we took of identifying profiles most at risk and accounting for uncertainty is of great value to policymakers interpreting our findings, such that they might consider how to provide targeted interventions aimed at reducing the overall burden of disease as well as disparities between area profiles.

A further strength of our study was that we were able to estimate suicide risk according to the date of actual death and not the registered date. Registration delay (sometimes up to years) is a common issue in official suicide statistics (for example those issued by ONS), as any unexpected deaths (including suspected suicides) involve a coroner's investigation to establish an official cause of death. Due to this delay approximately half of all unexpected deaths registered in a given year occurred in the previous year.^21^

Limitations of our study include that we were unable to account explicitly for some important individual (person) level factors of suicide, including ethnicity, income, unemployment, history of mental health problems, substance abuse or other adverse life events. Nonetheless, our use of the English IMD includes these factors implicitly to capture overall levels of deprivation. Instead of using area-level measure of employment and health, we used the IMD which is a composite score. Due to the high correlation between each IMD domains (see Supplementary Material), the use of the IMD meant we could capture all the aspects of deprivation implicitly rather than having to make a choice a priori and include only a few.

We also acknowledge that some of our area-level factors may have been inadequate proxies for the socio-environmental factor of interest for example the use of night-time light as light pollution. Given the ecological nature of the study, we could not make causal claims as well as misclassification and ecological bias may have been present. For example, we could not be sure that individuals who died by suicide in each area were subject to that area's socio-environmental characteristics. However, our use of a high-resolution spatial field limited the impact of these misclassification issues and biases. We used ten-year age groups and performed an age-sex standardisation which induces ecological bias, assumes the age-sex rates to be constant across the entire study period and temporal domain, and does not allow for explicit time and space varying age and sex interpretations. Both the grouping and standardisation were made to address the small counts, whilst the age-sex standardisation adjusted (implicitly) for age and sex and allowed for meaningful comparisons between geographical areas with different population structures.

Finally, we did not include data from Wales in our models as the English IMD is not directly comparable to the Welsh equivalent measure of deprivation. Suicide rates in Wales are known to be rising more quickly than in comparison to parts of England,^14^ and future studies should identify the extent to which various socio-environmental factors of mental health contribute to these national differences.

The findings we present here have important public mental health implications. Policy milestones over this period included the publication of the first suicide prevention strategy for England in 2002^39^ and a revised strategy in 2012.^40^ Both combined universal approaches with complementary high-risk targeted prevention interventions. Our data suggested that suicide risk in England fell sharply after the introduction of the 2002 suicide prevention strategy and remained at relatively low levels over the next decade. The 2012 revision was published immediately before a period of rises in risk from around 2014 onwards. Inferences about the effectiveness of the suicide prevention strategy over the latter part of our study period should be cautious for several reasons.^41^

First, our findings must be viewed within the broader context of England's mental health crisis. Since the early 21st century, the incidence of common mental disorders (CMD), which have a strong association with suicide,^42^^,^^43^ in English primary care has risen by 42.4%,^44^ equating to an estimated 1.1 million additional cases of depression and anxiety among England's 46 million residents aged 16+.^44^ Second, since the 2012 revision of England's suicide prevention strategy, several global (e.g., COVID-19, the Russian invasion of Ukraine) and national (e.g., the 2012 health service restructure, Brexit, the 2022 budget crisis, the cost-of-living crisis) events as well as the escalation of the climate crisis, which are likely negatively impacted population mental health, occurred. Third, there is plausible reason to believe that the shocks and climate crisis could have disproportionately affected the mental health of younger people, whose mental health has declined more sharply than others,^44^ who may be more vulnerable to the negative ramifications of these events, and who may have had less time to develop appropriate coping strategies to mitigate their effects on mental health.^45^ In light of these points, the absence of a corresponding rise in suicide rates may suggest the effectiveness of England's suicide prevention strategy. This is particular noteworthy in the context of COVID-19 where there was not the increase in suicides as expected.^38^^,^^46^ Whilst we do not know the counterfactual scenario under which rates would have changed in the absence of a suicide prevention strategy during this period, it is conceivable to suggest that rates may have risen even more rapidly. A final word of caution is that the national and regional trends presented in this Manuscript are dependent upon using the ICD-10 codes X60–X84, Y10–Y34 (excluding Y33.9), Y87.0, and Y87.2 as subsets of these change the definition and trend in suicide risk (see Supplementary Material).

Our study also informs the implementation of the research recommendations of the 2023 revision of the suicide prevention strategy for England to improve prevention through access to “timely and high-quality data, evidence and intelligence”.^19^ The identification of risk and protective socio-environmental factors aids the provision of “better understanding of the drivers of suicide and self-harm, the development of more effective interventions, and more rapid responses to prevent suicides”. Furthermore, the results exploring the full socio-environmental profiles provides aid in the same vein by taking a more holistic understanding of the complete influence of all included risk factors for suicide.

We found no substantial change in the overall risk of suicide in England from 2002 to 2022 but revealed strong spatio-temporal associations between suicide rates and various socio-environmental factors of health. In particular, our work highlighted small area community profiles in England that experienced higher suicide rates; these communities included more deprived, socially isolated, and rural areas, and those with greater density of road/rail networks and less greenspace. These findings, from high spatial resolution data, might be used to inform suicide prevention strategy nationally and target specifically regions characterised by high deprivation and rurality.

## Contributors

CG, MB, and BD were responsible for data acquisition. CG and MB had access to the raw data and were responsible for validation. CG and MB were responsible for the study conception, design and methodology. CG was responsible for the formal analysis, investigation, and visualisation. MB supervised the study. CG, JBK, and AP was responsible for writing the original draft. All authors contributed to reviewing and editing. SG, GB, JBK and MB were responsible for funding acquisition. SG, GB, JBK, MB, and BD were responsible for project administration and resourcing. CG was responsible for the decision to submit and subsequent revisions.

## Data sharing statement

The mortality and population data used in this work were supplied by the Office for National Statistics (ONS), derived from the national mortality registrations and the Census. SAHSU does not have permission to supply data to third parties.

Simulated data, code, and additional, animated plots can be found on the following GitHub repository: https://github.com/connorgascoigne/englishSuicides.

## Editor note

The Lancet Group takes a neutral position with respect to territorial claims in published maps and institutional affiliations.

## Declaration of interests

CG, BD, and MB acknowledge Infrastructure support for the Department of Epidemiology and Biostatistics provided by the NIHR Imperial Biomedical Research Centre (BRC). This paper does not necessarily reflect the views of UKHSA, NIHR or the Department of Health and Social Care.

AP and JK are supported by the NIHR University College Hospital London (UCLH) Biomedical Research Centre (BRC). AJ and JK are supported by UK Research and Innovation funding for the Population Mental Health consortium (grant no MR/Y030788/1) which is part of Population Health Improvement UK (PHI-UK), a national research network which works to transform health and reduce inequalities through change at the population level.

## Appendix A Supplementary data

Supplementary Materials

MSOA11 DIVERSITY

MSOA11 IMD

MSOA11 NDVI

MSOA11 NIGHTTIME LIGHT

MSOA11 POPULATION DENSITY

CORRELATION MATRIX
