# Supplementary material for: Spatio-temporal trends and socio-environmental determinants of suicides in England (2002–2022): an ecological population-based study
Source: Lancet Reg Health Eur. 2025 Aug 14;56:101386. doi: 10.1016/j.lanepe.2025.101386 (PMC12859596; doi:10.1016/j.lanepe.2025.101386)
Supplement: Supplementary Materials [file mmc1.pdf]

Supplementary Material for "Spatio-temporal trends and socio-environmental determinants of suicides in England (2002 – 2022): an ecological population-based study"

Connor Gascoigne[1*], Annie Jeffery[2], Ioannis Rotous[3], Xuewen Yu[4], Sara Geneletti[4], Bethan Davies[1, 5], Gianluca Baio[3], James B. Kirkbride[2], Alexandra Pitman[2], and Marta Blangiardo[1]

[1]MRC Centre for Environment and Health, Department of Epidemiology and Biostatistics, School of Public Health, Imperial College London, London, UK
[2]Division of Psychiatry, University College London, London, UK
[3]Department of Statistical Science, University College London, London, UK

[4]Department of Statistics, London School of Economics and Political Science, London, UK
[5]Small Area Health Statistics Unit (SAHSU), Department of Epidemiology and Biostatistics, School of Public Health, Imperial College London, London, UK
[*]Corresponding author: Connor Gascoigne, MRC Centre for Environment and Health, Department of Epidemiology and Biostatistics, School of Public Health, Imperial College London, London, UK. *E-mail address*: c.gascoigne@imperial.ac.uk

June 25, 2025

1 Socio-environmental factors

1.1 Data sources

To investigate key socio-environmental factors associated with suicide risk, we considered the following seven area-level factors at MSOA–level.

Deprivation [1, 2] was measured through the English Index of Multiple Deprivation (IMD) score, a composite score based on 39 separate indicators aggregated in seven domains [3]. IMDs were calculated in 2004, 2007, 2010, 2015 and 2019 over the study period. We attributed the IMD scores as follows: IMD2004 for 2002 – 2006, IMD2007 for 2007 – 2009, IMD2010 for 2010 – 2014, IMD2015 for 2015 – 2018, and IMD2019 for 2019 – 2022, respectively.

Ethnic density [4–6] was derived as the proportion of people from minoritised ethnic backgrounds per MSOA [7]. This was based on the ethnicity population totals released by the ONS at the Censuses in 2001, 2011 and 2021. The 2001 census data were applied to the period 2002 – 2005, the 2011 census data to 2006 – 2015, and the 2021 census data to 2016 – 2022.

Population density [8, 9] was calculated as the total population of each MSOA [10] divided by the total area (km^2) of that MSOA.

Light pollution [11] was captured with a measure of night-time light using the harmonized global night-time light dataset from satellite images [12]. The data were downloaded as a yearly 1km $\times$ 1km raster (gridded pixel) and aggregated (by a weighted average) to the MSOA level.

Railway network density [13, 14] was captured using railways network data downloaded from OpenStreetMap [15]. The MSOA-level railway network density was captured by dividing the total length (km) of railway in each MSOA by the total area (km^2) of the MSOA.

Road network density [13, 14] was captured using the same approach as for railway network density but the data was sourced from the Ordnance Survey Open Map [16].

Greenspace [17, 18] was captured using the Normalized Difference Vegetation Index (NDVI). This was downloaded from NASAs moderate resolution imaging spectroradiometer satellite as a 16-day 250m $\times$ 250m gridded raster [19]. The NDVI was aggregated into years and MSOAs using an average and weighted average, respectively.

1.2 Data sorting

In the UK, a census occurs every ten years with three occurring across our study period; 2001, 2011, and 2021. After a census, the UKs Office for National Statistics (ONS) may update the administrative area boundaries based on the new population structure to ensure a consistent population. For example, the Lower layer Super Output Area (LSOA) and Middle layer Super Output Area (MSOA) should contain between 400 – 1,200 and 5,000 – 15,000 individuals, respectively. An MSOA is a collection of mutually exclusive and collectively exhaustive LSOAs (i.e., there is a many-to-one mapping of LSOAs-to-MSOAs). For our analysis, we used the MSOA boundaries defined by the 2011 census (MSOA11). The ONS provides both the boundaries and documents to map between the boundaries. The mapping included links between difference administrative units for the same census (i.e., LSOA11-to-MSOA11), and between census' for the same administrative unit (i.e., LSOA01-to-LSOA11) [20].

In our study, the data downloaded from satellite sources (light pollution and greenspace), were mapped directly onto the MSOA11 boundaries using a weighted average. The railway network and road network densities were also mapped directly to the MSOA11 boundaries. The data for deprivation (derived from IMD score), ethnic density (derived from ethnic population totals), and population density (derived from full population totals) were downloaded at the LSOA administrative unit. The specific census boundaries used depended on the year of the data. Table 1 presents which census is applied to which IMD score, ethnicity population total, and full population total release.

To map the IMD score, ethnicity-, and full-population totals onto the MSOA11 boundaries, we performed two steps 1) map all releases onto the LSOA11 (i.e., LSOA01-to-LSOA11, and LSOA21-to-LSOA11) boundary, and 2) map the LSOA11 data onto the MSOA11 boundary (i.e, LSOA11-to-MSOA11). Due to an increase in the number LSOAs after the 2011 census, after step 1) there were four occurrences of LSOAs defined at the 2011 census that were not present in the 2001 census. Namely: E01032522, Nottingham 028F; E01032540, Hartlepool 003G; E01032608, Leeds 067K; and, E01033730, Greenwich 035D. We imputed the missing values by taking an average of first-order (i.e., they share a direct, coterminous, border) neighbours. For step 2) we mapped the LSOA11-to-MSOA11 codes using a population weighted average for the IMD score and by summing the ethnicity-, and full-population totals. The population used to weight the IMD scores in Step 2) were the mid-year population totals for the year being aggregated. For example, we used the 2004 mid-year population totals to aggregate the 2004 IMD score (now on the 2011 LSOA structure) up the 2011 MSOA structure.

Table 1: Defining what Lower Layer Super Output Area (LSOA) structure is used by the Office for National Statistics releases for the LSOA level Index of Multiple Deprivation and ethnic population totals.

**LSOA structure**	**IMD Release**	**Ethnic Population Release**	**Full Population Release**
2001 Census	2004, 2007, 2010	2001	2002 – 2010
2011 Census	2015, 2019	2011	2011 – 2021
2021 Census	-	2021	2022

1.3 Visualisation

Deprivation, ethnic density, population density, light pollution, and greenspace were treated as time varying over the study period. Railway and Road network densities were not time varying. Maps of the average score for each socio-environmental factor (with the exception of railway network density and road network density as they do not vary in time) are presented in Figure 1. To understand the temporal dynamic of the socio-environmental factors, we have provided animated `.gif` files of the five time varying socio-environmental factors. These are provided on the GitHub https://github.com/connorgascoigne/englishSuicides.

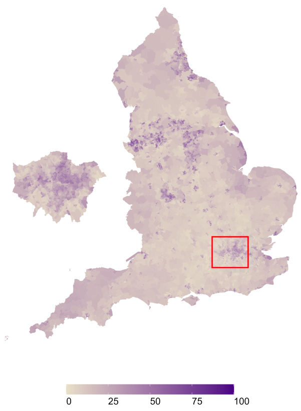

(a) Deprivation

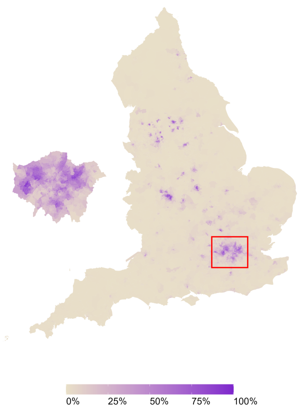

(b) Ethnic density

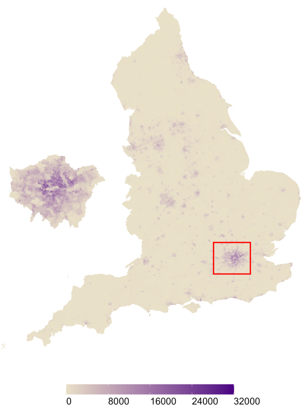

(c) Population Density

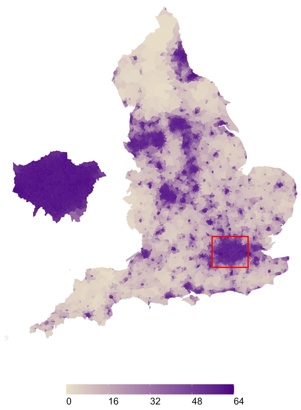

(d) Light Pollution

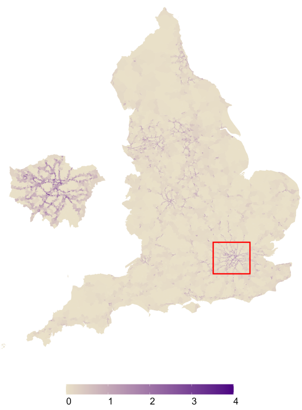

(e) Railway Network Density

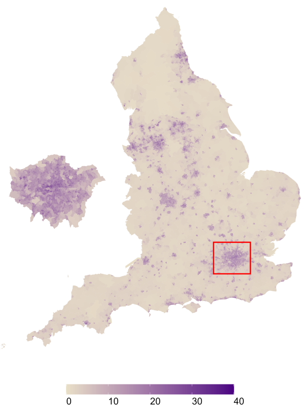

(f) Road Network Density

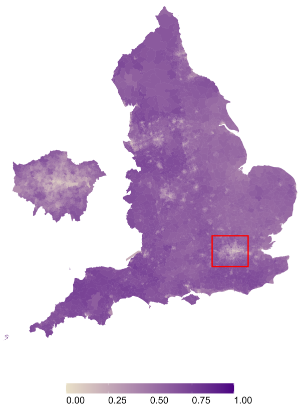

(g) Greenspace

Figure 1: Maps of the average (with the exception of railway network density and road network density) score for each socio-environmental factor over the study period (2002 – 2022).

1.4 Socio-environmental factor specification within the model

Table 2 presents how each socio-environmental factor was specified in the modelling process. All socio-environmental factors were standardised (i.e., subtracted the mean and divided by the standard deviation). Figure 2 shows histograms of the standardised socio-environmental scores.

Table 2: Environmental factors specification within the model.

**Socio-environmental Factor (unit)**	**Continuous Score**
Deprivation (value)	0 (less deprived) to 100
Ethnic density (%)	0% (no non-white population) to 100%
Population density (per KM^2)	0 (no population) to ∞
Light pollution (DN value)	0 (none) to 63
Railway network density (per KM^2)	0 (no railway network) to ∞
Road network density (per KM^2)	0 (no road network) to ∞
Greenspace (value)	0 (little-to-no vegetation) to 1

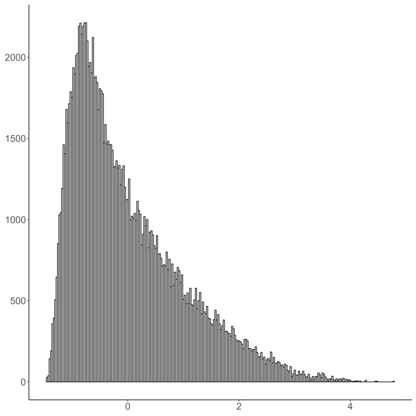

(a) Deprivation

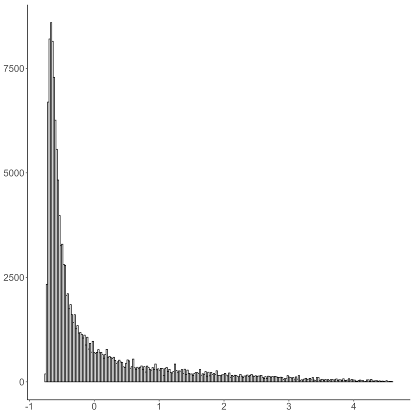

(b) Ethnic density

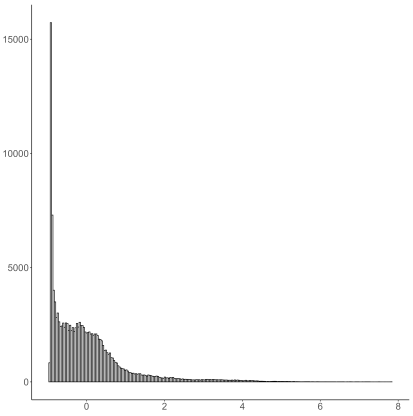

(c) Population Density

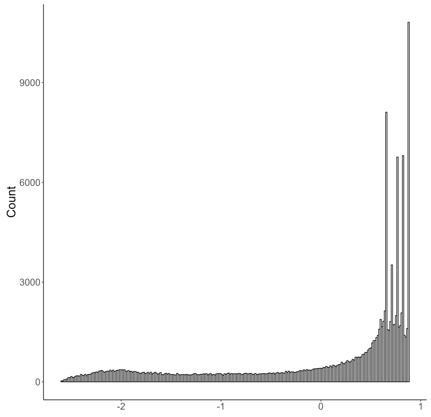

(d) Light Pollution

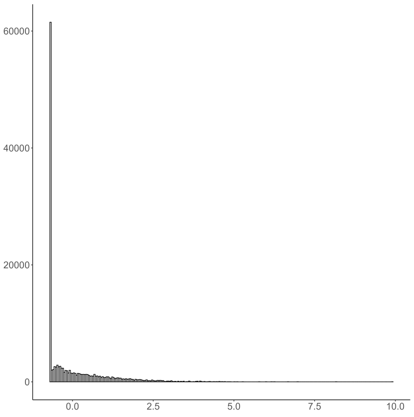

(e) Railway Network Density

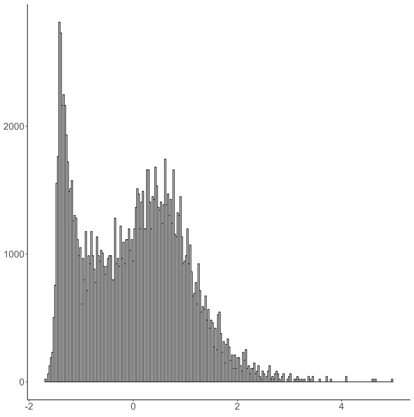

(f) Road Network Density

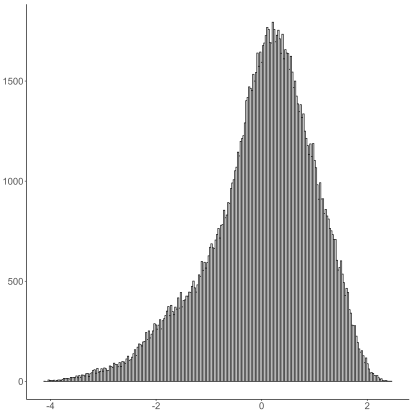

(g) Greenspace

Figure 2: Histograms of the standardised continuous scores over the study period (2002 – 2022) for each of the socio-environmental factors.

1.5 Correlation between socio-environmental factors

We measured the Pearson's correlation between the environmental factors and present the results in Figure 3. The largest correlation, 0.80, was between population density and road network density. The smallest correlation, 0.15, was between the railway network density and deprivation. One of population density or road network density could have been omitted due to the high, 0.80, correlation. However, we included all socio-environmental factors as we found all model estimates were stable, i.e., we did not have extremely large and unreasonable uncertainty intervals for any model parameters. To understand the temporal dynamic of the correlation, we have provided animated `.gif` files of the correlation matrix. This will be provided on the main authors' GitHub https://github.com/connorgascoigne/englishSuicides.

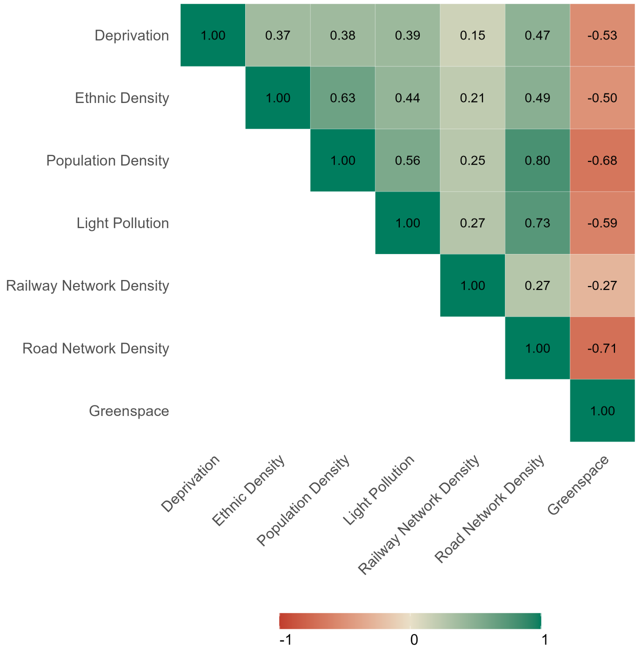

Figure 3: Correlation matrix for the socio-environmental factors.

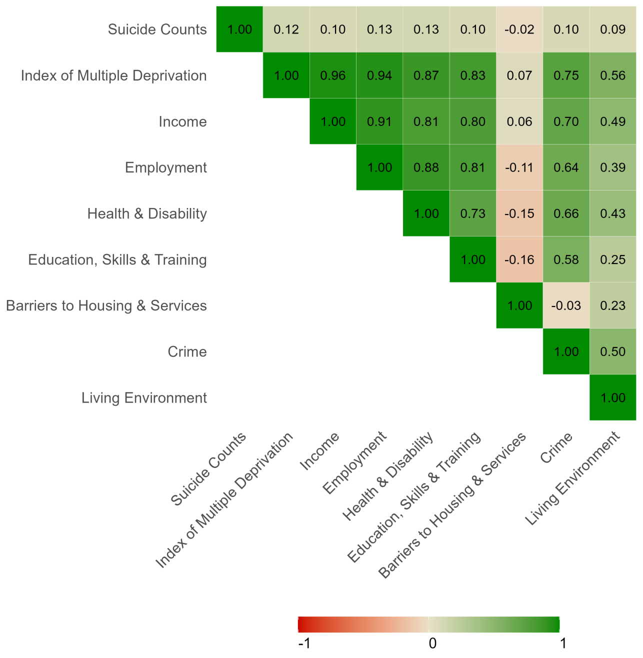

Figure 4: Correlation between suicide counts, the Index of Multiple Deprivation (IMD), and the seven domains that define the IMD. and each covariates over the study period.

1.6 Correlation within the Index of Multiple Deprivation

Figure 4 presents the correlation between suicide counts, the IMD, and the seven domains (Income, 22.5%; Employment, 22.5%; Health and Disability, 13.5%; Education, Skills and Training, 13.5%; Barriers to Housing and Services, 9.3%; Crime, 9.3%; and, Living Environment, 9.3%) that make up the IMD.

The Health and Disability domain contains a measure of suicide counts. We calculated the weighting of the measure of suicides used within the IMD [21]. The Health and Disability domain consists of four subdomains: i) years of potential life lost, ii) comparative illness and disability ratio, iii) acute morbidity, and iv) mood and anxiety disorders. The weighting for each subdomain was 27.1%, 30.0% 25.6%, and 17.2%, respectively. The latter subdomain (mood and anxiety disorders; 17.2%), was derived using data from hospital episodes, prescribing, and suicide mortality. The weighting for each dataset was 38.1%, 41.5%, and 20.5%, respectively. Based on these weights, the suicide aspect of the IMD accounts for $100 \times (0.135 \times 0.172 \times 0.205) = 0.48\%$ of the total IMD. To ensure this caused no issues in the analysis, we performed a correlation test between suicide counts and all seven domains (including the Health and Disability domain) of the IMD. The correlation between the suicide counts and both the IMD and the Health and Disability domain was 0.12 and 0.13, respectively, implying there will be no issues in using the IMD within the analysis.

The correlation between the IMD and five of the seven domains was extremely high (the correlation between IMD and Living Environment and Barriers to Housing and Services was mild, 0.56, and low, 0.07, respectively) implying they are capturing similar aspects of suicide risk. We used the IMD instead of the seven individual domains due to the other domains having a high correlation amongst themselves.

2 Model specification

2.1 Age-sex standardisation

We performed an age-sex standardisation to obtain adjusted expected counts for each MSOA-year in the study period. To perform the age-sex standardisation we followed three steps 1) define a reference 2) calculate the expected number of suicides, and 3) marginalise out the terms being standardised. Let Y_{asit} and N_{asit} be the number of suicides and total population in the ath age group, s^{th} sex, i^{th} MSOA, and t^{th} year. We performed the following:

1. Define the reference

Using the whole study domain (England) and study period (2002 - 2022), we calculated the age-sex specific suicide rates

$$R_{as} = \sum_{it} \frac{Y_{itas}}{N_{itas}}$$

which where were used as the reference.

2. Calculate the expected suicides

For each MSOA-year-age-sex combination, we calculated the expected number of suicides,

$$E_{iats} = N_{itas} \times R_{as}.$$

3. Marginalise out the terms being standardised

To complete the age-sex standardisation, we marginalised out (in this case by summing over) the age and sex stratification,

$$\begin{aligned} Y_{it} &= \sum_{as} Y_{itas} \\ E_{it} &= \sum_{as} E_{itas} = \sum_{as} N_{itas} \times R_{as}. \end{aligned}$$

2.2 Hurdle model

To account for the high number of zeros seen throughout our study period $\approx$ (61%), we modelled suicide counts using a Hurdle model [22–24]. A Hurdle model is a two component model where the zeros and (truncated) counts are assumed to come from two separate data generating mechanisms but are modelled together. Suitable distributions for the zero and count components are the binomial, or a censored count distribution, for the former and truncated Poisson, or truncated Negative Binomial, for the latter [25].

For $i = 1, \ldots, I = 6,791$ and $t = 2002, \ldots, T = 2022$, the indices for MSOA and year, let Y_{it} and E_{it} be the number of- and (age-sex adjusted) expected number of-suicides in the ith MSOA and t^{th} year. We defined $\boldsymbol{Y} = (\boldsymbol{Z}, \boldsymbol{O})$ where the probability of event y_{it} occurring is defined

$$\Pr(\boldsymbol{Y} = y_{it}) = \begin{cases} \Pr(\boldsymbol{Z} = y_{it}), & \text{if } y_{it} = 0 \\ [1 - \Pr(\boldsymbol{Z} = y_{it})] \times \frac{\Pr(\boldsymbol{O} = y_{it})}{1 - \Pr(\boldsymbol{O} = 0)}, & \text{if } y_{it} \geq 1 \end{cases}$$

where $\boldsymbol{Z}$ is a binary vector of whether the suicide has occurred or not, and $\boldsymbol{O}$ is a vector of the observed number of suicides. Alternatively,

$$z_{it} = \begin{cases} 1 & \text{if } y_{it} \neq 0 \\ 0 & \text{otherwise} \end{cases} \quad \text{and} \quad o_{it} = \begin{cases} NA & \text{if } y_{it} = 0 \\ y_{it} & \text{otherwise} \end{cases}.$$

An example of the form of the data expressed in general and with synthetic values is

$$\begin{bmatrix} z_{1,2002} & NA \\ z_{1,2003} & NA \\ \vdots & \vdots \\ z_{6791,2022} & NA \\ -- & -- \\ NA & o_{1,2002} \\ NA & o_{1,2003} \\ \vdots & \vdots \\ NA & o_{6791,2022} \end{bmatrix} \longrightarrow \begin{bmatrix} 1 & NA \\ 0 & NA \\ \vdots & \vdots \\ 1 & NA \\ -- & -- \\ NA & 7 \\ NA & NA \\ \vdots & \vdots \\ NA & 3 \end{bmatrix}.$$

In our specification of the Hurdle model, we used a binomial distribution for the zero component and a truncated Poisson distribution for the count component. We refer to this as a Hurdle Poisson (HP) model. Under the HP specification, suicides were modelled as

$$Y_{it} \sim \text{HurdlePoisson}\left(\pi_{it}, \rho_{it} E_{it}\right)$$

where π_{it} and ρ_{it} were the mean functions for the zero and truncated count components of the model, and E_{it} is the expected number of suicides. The HP model can be written as

$$\begin{aligned} \text{logit}\left(\pi_{it}\right) &= \beta_0^z + \boldsymbol{X}\boldsymbol{\beta}^z + \delta_i + \gamma_t + \xi_{it} \\ \log\left(\rho_{it}\right) &= \log\left(E_{it}\right) + \beta_0^o + \boldsymbol{X}\boldsymbol{\beta}^o + \beta_\delta^o \delta_i + \beta_\gamma^o \gamma_t + \beta_\xi^o \xi_{it} \end{aligned}$$

where logit (π_{it}) was the linear predictor for the zero component and log (ρ_{it}) was the linear predicted for the truncated counts.

In the two linear predictors, β_0^z and β_0^o were the zero- and count-component specific intercept and the terms $\boldsymbol{\beta}^z$ and $\boldsymbol{\beta}^o$ were the component specific regression coefficients for the socio-environmental factors. The terms δ_i, γ_t, and ξ_{it} were the temporal, spatial and spatio-temporal random effect parameters, respectively, accounting for any residual confounding in each scale. As the spatial, temporal and spatio-temporal random effects were shared across the two components, the parameters β_δ^o β_γ^o, and β_ξ^o were included in the count components linear predictor to account for the difference scales of the two distributions. We called these the 'scale' parameters.

2.3 Sampling

Sampling from a HP model required the combination of a Bernoulli distribution with a truncated Poisson distribution in a non-trivial way. Here we provide an explanation of how this is done.

1. Generate a sample from a Bernoulli distribution:

   $$\hat{z}_{it} \sim \text{Binomial}\left(n = 1, \pi = \hat{\pi}_{it}\right)$$

   where $\hat{\pi}_{it}$ was estimated from the zero components linear predictor.

2. Generate samples from truncated Poisson distribution:

   $$\hat{y}_{it} = \begin{cases} 0, & \text{if } \hat{z}_{it} = 0 \\ \hat{o}_{it} \sim \text{zt-Poisson}\left(\hat{\rho}_{it} E_{it}\right), & \text{if } \hat{z}_{it} = 1 \end{cases}$$

   where zt stands for 'zero-truncated' and $\hat{\rho}_{it}$ is estimated from the count components linear predictor.

2.4 Prior specification

Fixed effects

For all fixed effects, we used weakly informative normal priors. Namely for the intercepts, $\beta_0^z, \beta_0^o \sim \text{Normal}\left(0, +\infty\right)$. For the regression coefficients, $\boldsymbol{\beta}^z, \boldsymbol{\beta}^o \sim \text{Normal}\left(0, 1000\right)$. For the scale parameters, β_δ^o $\beta_\gamma^o, \beta_\xi^o \sim \text{Normal}\left(1, 10\right)$. For the scale parameter, the prior was centered about 1 as this referred to no scaling.

Spatial random effects

We modelled the spatial random effect using a weighted average of an unstructured and structured component. The unstructured component was an exchangeable model, and the structured component was defined by an autoregressive model specified by the neighbourhood structure of the spatial domain. This ensures the spatial model was flexible enough to provide both global (unstructured) and local (structured) smoothing [26, 27]. The spatial random effect was,

$$\delta_{s(i)} = \frac{1}{\sqrt{\tau_\delta}} \left(\sqrt{\phi} u^*_{s(i)} + \sqrt{1-\phi} v^*_{s(i)} \right),$$

where $u^*_{s(i)}$ and $v^*_{s(i)}$ were standardised versions of the structured and unstructured components, respectively, to have variance equal to one, $\phi \in [0,1]$ was the mixing parameter which attributes how much of the residual spatial variation was due to the structured component, and τ_δ was the precision which was equal to the inverse of the variance, i.e., $\sigma^2_\delta = 1/\tau_\delta$.

Temporal random effects

We modelled the temporal random effect using a first order random walk (RW1) [28],

$$\gamma_t \propto \tau_\gamma^{(T-1)/2} \exp \left(-\frac{\tau_\gamma}{2} \sum_{t=1}^{T-1} (\Delta\gamma_t)^2 \right),$$

where $\delta\gamma_t = \gamma_{t+1} - \gamma_t$ was the first order difference between pairs of γ. The RW1 prior smooths locally towards the first order temporal neighbours.

spatio-temporal random effects

The spatio-temporal random effect was modelled using a normal prior with zero mean and a given standard deviation [29, 30],

$$\xi_{it} \sim \text{Normal}\left(0, \tau_\xi\right).$$

For each of the spatial and temporal components of the spatio-temporal term, we assumed they were both unstructured.

2.5 Hyperparameters

We specified penalised complexity (PC) priors [27, 31] on the standard deviation and mixing parameter components. A PC prior for a given model component is specified through $\Pr(\text{model parameters} > U) = p$ where U is an appropriate upper bound for the distribution of the parameter and p is the probability of the model parameter being in the upper bound.

All the precision parameters (spatial, τ_δ, temporal, τ_γ, and spatio-temporal, τ_ξ) had a PC prior where $U = 1$ and $p = 0.01$. This reflected the prior belief that the spatial, temporal and spatio-temporal terms were not likely to affect the observed relationship. The spatial effect mixing parameter, ϕ, had a $\Pr(\phi > 1/2) = 2/3$ PC prior, where p was larger than 0.5 to reflect the prior belief for more of the spatial variation to be described by the unstructured spatial effect.

3 Model parameters

Table 3 shows the full set of model parameters for all components of the HP model. There are three main sections 1) the regression coefficients 2) the random effects and 3) the variability explained by each set of parameters. We let the socio-environmental factors be independent when modelling the zero and count components and have regression coefficients for both the zero and count components of the HP model. For each random effect, we shared these between the zero and count components, and had one precision parameter and scale parameter.

Regression coefficients

The interpretation of the regression coefficients differs for each component; the zero component coefficients are odds ratios and the count component coefficients are relative risk. For example, the zero component coefficients describe how much the odds of at least one suicide occurring would change when comparing to the reference level for the factor, whereas the count component coefficients describe how much the relative risk of suicide changes when comparing against the reference category of the factor, assuming that at least one suicide has occurred.

In the Manuscript, we presented the change in the Relative Risk (RR) of a difference of one standard deviation. There we presented substantial positive associations between suicide and deprivation, railway network density, and road network density. Furthermore, we presented substantial negative associations between suicide and ethnic density, population density, light pollution and greenspace. Looking at the Binomial and Poisson regression coefficients in Table 3, the effect sizes for all parameters are the same.

Random effects

We first considered the precision for each parameter. A larger precision (or alternatively, a smaller variance) indicated the parameter was not explaining a lot of variation with the converse being true for a smaller precision. Therefore, the spatial random effect, with the smallest precision, wad the most influential of the random effects, and the spatio-temporal random effect was mildly influential in comparison and the temporal random effect was less influential. The spatial mixing parameter indicated 60.36% (95% Credible Interval (CrI): 52.01%, 68.05%) of the spatial variability was explained explicitly by the neighbourhood structure.

Variability explained

As described in the Manuscript, the socio-environmental factors and spatial, temporal, and spatio-temporal random effects explain 38.95% (95% CrI: 34.54%, 43.40%), 55.13% (95% CrI: 49.86%, 60.10%), 5.51% (95% CrI: 4.58%, 6.61%), and 11.67% (95% CrI: 5.53%, 18.74%), respectively, of the total variation we saw in suicides. There was a proportional relationship between the precision estimates and the variability explained, where a smaller precision was equivalent to a larger percentage explained.

Table 3: All parameters from the Hurdle Poisson model. The parameters are split into either the zero or counts components. For parameters which are shared, they are across to the two columns for the zero and count.

	**Zero Component**	**Count Component**
**Intercept:**	-0.07971 (-0.09195, -0.06747)	-0.05105 (-0.07222, -0.03201)
**Deprivation:**	0.24881 (0.2307, 0.26694)	0.19605 (0.18241, 0.20972)
**Ethnic Density:**	-0.09067 (-0.11324, -0.06807)	-0.09666 (-0.11347, -0.07984)
**Population Density:**	-0.05812 (-0.09116, -0.02507)	-0.07402 (-0.09736, -0.05071)
**Light Pollution:**	-0.05802 (-0.08031, -0.03569)	-0.03936 (-0.05772, -0.02094)
**Railway Network Density:**	0.02548 (0.01121, 0.03975)	0.01124 (-0.00098, 0.02346)
**Road Network Density:**	0.0659 (0.03383, 0.09794)	0.06296 (0.03817, 0.08773)
**Greenspace:**	-0.10332 (-0.12676, -0.07989)	-0.04375 (-0.06142, -0.02609)
**Spatial Random Effects:**		
Spatial mixing	0.60357 (0.52012, 0.6805)	
Precision	5.87399 (5.27901, 6.52436)	
Scale	0.47715 (0.43257, 0.52299)	
**Temporal Random Effects:**		
Precision	228.19163 (134.93383, 603.18605)	
Scale	0.43768 (0.33742, 0.53815)	
**spatio-temporal Random Effects:**		
Precision	85.94641 (47.07907, 165.39173)	
Scale	1.42572 (0.95891, 1.85942)	
**Variability Explained: (%)**		
Socio-environmental factors	38.9514% (34.5441%, 43.4029%)	
Spatial term	55.1265% (49.864%, 60.0993%)	
Temporal term	5.5053% (4.5772%, 6.6084%)	
spatio-temporal term	11.6724% (5.5278%, 18.7386%)	

4 Sensitivity Analysis

To be consistent with English literature on suicides [32–34], we defined suicide deaths using the following ICD-10 codes X60 – X84, Y10 – Y34 (excluding Y33.9), Y87.0, and Y87.2. For this section only, we refer to these as the $X^\star$ (intentional self-harm) and $Y^\star$ (events of undetermined intent) codes, respectively.

In 2011, there was a change to how deaths were coded that had the potential to influence suicide numbers. For full details, see the 2011 bulletin [32]. In brief, prior to 2011, deaths where the coroner's report mention both acute poisoning and drug dependence would be assigned an underlying cause of a mental and behavioural disorder due to psychoactive substance use. Consequently, they would be given an ICD-10 'F' code and not be included in suicide statistics. Post 2011, these deaths would be coded as either an accidental poisoning (ICD-10 code: X40 – X49), or a poisoning of undermined intent (ICD-10 code: Y10 – Y19). The latter case would potentially increase the number of deaths included in suicide statistics via the $Y^\star$ codes with the $X^\star$ codes unaffected. Based on an analysis on the 2011 statistics, the ONS found no change in deaths for the $X^\star$ codes and a 2% increase in deaths for the $Y^\star$ codes for England and Wales. In Scotland, there was an increase in the number of suicides via the $Y^\star$ codes.

As our methods differ from those of the ONS, we performed a sensitivity analysis using the $X^\star$ codes only. This included recalculating the age-sex standardised expected number of suicides based on only the $X^\star$ codes over the study period and domain. We reproduced the national and regional RR over the study period as well as the percentage change in the risk of suicide for a one standard deviation increment for each socio-environmental factor (adjusted for average effect of the six other socio-environmental factors as well as space and time). The results are presented in Figure 5.

In Figure 5, the national trends (panel (a)) in risk rose from 0.92 (95% CrI: 0.89, 0.96) in 2002 to 1.15 (95% CrI: 1.11, 1.18) in 2022. This was a substantial increase of 23.8% (95% CrI: 17.6%, 30.2%). All regional trends (panel (b)) showed an increasing risk over the study period, similar to the national trend. Regarding the percentage change in suicide risk associated with a one standard deviation increase in each of the seven socio-environmental factors: deprivation, railway network density, and road network density were identified as risk factors; whilst ethnic density, population density, light pollution, and greenspace were identified as protective factors.

Comparing between the results in the Manuscript (using both $X^\star$ and $Y^\star$ codes) to those in the sensitivity analysis (using $X^\star$ codes only), the interpretations of the seven socio-environmental covariates do not change. The interpretations of the national and regional relative risk do change. In the Manuscript, the interpretation of the national risk from the start to the end of the study period was a non-substantial decrease. In the sensitivity analysis, this was a substantial increase. Consequently, the discussion regarding the effectiveness of the 2012 suicide prevention scheme [35] is sensitive to the choice of suicide code used. Based on the results of the sensitivity analysis, we hypothesis reductions in the risk of suicide are due to reductions in the risk of death by undetermined intent. Further investigation into these differences is left for future work.

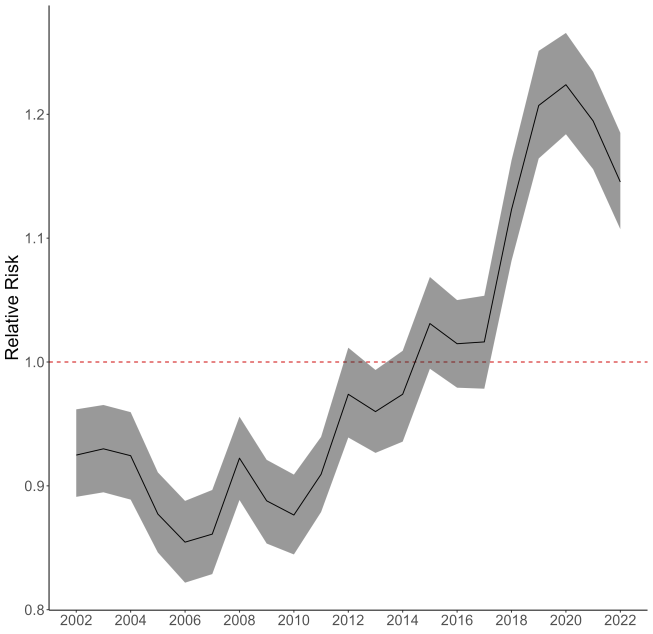

(a) National RR.

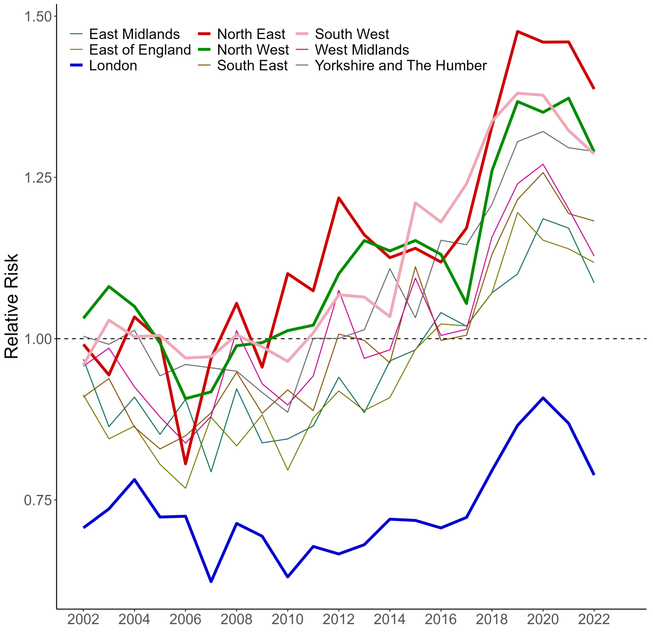

(b) Regional RR.

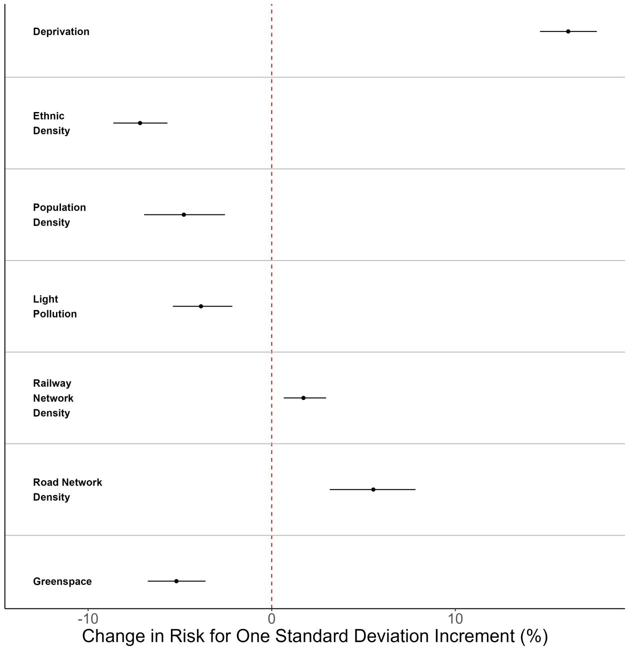

(c) Δ% in risk for 1SD increment.

Figure 5: Results of the sensitivity analysis where only ICD-10 Codes X60 – X84 were used. We reproduced the national (top-left) and regional (top-right) Relative Risk (RR) over the study period. Additionally, we reproduced the percent change (Δ%) in the risk for One Standard Deviation (1SD) increment in each of the socio environmental factors from the adjusted model (bottom-centre).

References

[1] Nicos Middleton, Jonathan AC Sterne, and David Gunnell. The geography of despair among 15–44-year-old men in England and Wales: putting suicide on the map. *Journal of Epidemiology & Community Health*, 60(12): 1040–1047, 2006.

[2] Peter Congdon. Explaining the spatial pattern of suicide and self-harm rates: A case study of east and south east England. *Applied Spatial Analysis and Policy*, 4:23–43, 2011.

[3] Office for National Statistics. English indices of deprivation, 2020. URL https://www.gov.uk/government/collections/english-indices-of-deprivation. Last accessed 01 January 2025.

[4] Danah Alothman, Andrew Fogarty, Edward Tyrrell, Sarah Lewis, and Timothy Card. Ethnicity and suicide risk: A population-based study from England. *Journal of Affective Disorders*, 298:555–557, 2022.

[5] Duleeka Knipe, Paul Moran, Laura D Howe, Saffron Karlsen, Nav Kapur, Lauren Revie, and Ann John. Ethnicity and suicide in england and wales: a national linked cohort study. *The Lancet Psychiatry*, 11(8):611–619, 2024.

[6] Isobel L Ward, Katie Finning, Daniel Ayoubkhani, Katie Hendry, Emma Sharland, Louis Appleby, and Vahé Nafilyan. Sociodemographic inequalities of suicide: a population-based cohort study of adults in England and Wales 2011–21. *European Journal of Public Health*, 34(2):211–217, 2024.

[7] Office for National Statistics. Nomis, 2025. URL https://www.nomisweb.co.uk/. Last accessed 01 January 2025.

[8] Marco Helbich, Paul L Plener, Sebastian Hartung, and Victor Blüml. Spatiotemporal suicide risk in Germany: A longitudinal study 2007–11. *Scientific Reports*, 7(1):7673, 2017.

[9] Sasikiran Kandula, Gonzalo Martinez-Alés, Caroline Rutherford, Catherine Gimbrone, Mark Olfson, Madelyn S Gould, Katherine M Keyes, and Jeffrey Shaman. County-level estimates of suicide mortality in the USA: a modelling study. *The Lancet Public Health*, 8(3):e184–e193, 2023.

[10] Office for National Statistics. Population estimates for the uk, england, wales, scotland and northern ireland statistical bulletins, 2023. URL https://www.ons.gov.uk/peoplepopulationandcommunity/populationandmigration/populationestimates/bulletins/annualmidyearpopulationestimates/previousReleases. Last accessed 01 January 2025.

[11] Angus C Burns, Daniel P Windred, Martin K Rutter, Patrick Olivier, Céline Vetter, Richa Saxena, Jacqueline M Lane, Andrew JK Phillips, and Sean W Cain. Day and night light exposure are associated with psychiatric disorders: an objective light study in $>$ 85,000 people. *Nature Mental Health*, 1(11):853–862, 2023.

[12] Qingling Zhang and Karen C Seto. Mapping urbanization dynamics at regional and global scales using multi-temporal dmsp/ols nighttime light data. *Remote Sensing of Environment*, 115(9):2320–2329, 2011.

[13] Benedikt Wicki, Beat Schäffer, Jean Marc Wunderli, Thomas J Müller, Charlotte Pervilhac, Martin Röösli, and Danielle Vienneau. Suicide and transportation noise: a prospective cohort study from Switzerland. *Environmental Health Perspectives*, 131(3):037013, 2023.

[14] Guang Hao, Lei Zuo, Peng Xiong, Li Chen, Xiaohua Liang, and Chunxia Jing. Associations of pm2. 5 and road traffic noise with mental health: Evidence from uk biobank. *Environmental Research*, 207:112221, 2022.

[15] OpenStreetMap. Openstreetmap, 2024. URL https://www.openrailwaymap.org/. Last accessed 01 January 2025.

[16] Ordnance Survey. Os open roads, 2024. URL https://osdatahub.os.uk/downloads/open/OpenRoads. Last accessed 01 January 2025.

[17] Marco Helbich, Derek De Beurs, Mei-Po Kwan, Rory C O'Connor, and Peter P Groenewegen. Natural environments and suicide mortality in the Netherlands: a cross-sectional, ecological study. *The Lancet Planetary Health*, 2(3):e134–e139, 2018.

[18] Hilbert Mendoza, Lucía Rodriguez-Loureiro, Sylvie Gadeyne, Wouter Lefebvre, Charlotte Vanpoucke, and Lidia Casas. Urban green spaces and suicide mortality in Belgium (2001–2011): a census-based longitudinal study. *Environmental Research*, 216:114517, 2023.

[19] National Aeronautics and Space Administration. Modis vegetation index products (ndvi and evi), 2024. URL https://modis.gsfc.nasa.gov/data/dataprod/mod13.php. Last accessed 01 January 2025.

[20] Office for National Statistics. The open geography portal, 2023. URL https://geoportal.statistics.gov.uk/. Last accessed 01 January 2025.

[21] Ministry of Housing, Communities and Local Government. English indices of deprivation 2019: technical report, 2019. URL https://www.gov.uk/government/publications/english-indices-of-deprivation-2019-technical-report. Last accessed 01 June 2025.

[22] John Mullahy. Specification and testing of some modified count data models. *Journal of Econometrics*, 33(3): 341–365, 1986.

[23] David C Heilbron. Zero-altered and other regression models for count data with added zeros. *Biometrical Journal*, 36(5):531–547, 1994.

[24] Cindy Xin Feng. A comparison of zero-inflated and hurdle models for modeling zero-inflated count data. *Journal of Statistical Distributions and Applications*, 8(1):8, 2021.

[25] Achim Zeileis, Christian Kleiber, and Simon Jackman. Regression models for count data in R. *Journal of Statistical Software*, 27(8):1–25, 2008.

[26] Julian Besag, Jeremy York, and Annie Mollié. Bayesian image restoration, with two applications in spatial statistics. *Annals of the institute of Statistical Mathematics*, 43:1–20, 1991.

[27] Andrea Riebler, Sigrunn H Sørbye, Daniel Simpson, and Håvard Rue. An intuitive Bayesian spatial model for disease mapping that accounts for scaling. *Statistical Methods in Medical Research*, 25(4):1145–1165, 2016.

[28] Håvard Rue, Sara Martino, and Nicolas Chopin. Approximate Bayesian inference for latent Gaussian models by using integrated nested Laplace approximations. *Journal of the Royal Statistical Society Series B: Statistical Methodology*, 71(2):319–392, 2009.

[29] Leonhard Knorr-Held. Bayesian modelling of inseparable space-time variation in disease risk. *Statistics in medicine*, 19(17-18):2555–2567, 2000.

[30] Marta Blangiardo and Michela Cameletti. *Spatial and spatio-temporal Bayesian models with R-INLA*. John Wiley & Sons, 2015.

[31] Daniel Simpson, Håvard Rue, Andrea Riebler, Thiago G Martins, and Sigrunn H Sørbye. Penalising model component complexity: A principled, practical approach to constructing priors. *Statistical Science*, 32:1–28, 2017.

[32] Office for National Statistics. Suicides in england and wales statistical bulletins, 2024. URL https://www.ons.gov.uk/peoplepopulationandcommunity/birthsdeathsandmarriages/deaths/bulletins/suicidesintheunitedkingdom/previousReleases. Last accessed 01 January 2025.

[33] Cathryn Rodway, Saied Ibrahim, Jodie Westhead, Lana Bojanić, Pauline Turnbull, Louis Appleby, Andy Bacon, Harriet Dale, Kate Harrison, and Nav Kapur. Suicide after leaving the UK Armed Forces 1996–2018: A cohort study. *PLoS Medicine*, 20(8):e1004273, 2023.

[34] Navneet Kapur, David While, Nick Blatchley, Isabelle Bray, and Kate Harrison. Suicide after leaving the uk armed forces—a cohort study. *PLoS medicine*, 6(3):e1000026, 2009.

[35] Department of Health and Social Care. Suicide prevention strategy for england, 2012. URL https://www.gov.uk/government/publications/suicide-prevention-strategy-for-england. Last accessed 01 January 2025.
