# Supplementary figures and images for: Spatio-temporal trends and socio-environmental determinants of suicides in England (2002–2022): an ecological population-based study
Source: Lancet Reg Health Eur. 2025 Aug 14;56:101386. doi: 10.1016/j.lanepe.2025.101386 (PMC12859596; doi:10.1016/j.lanepe.2025.101386)

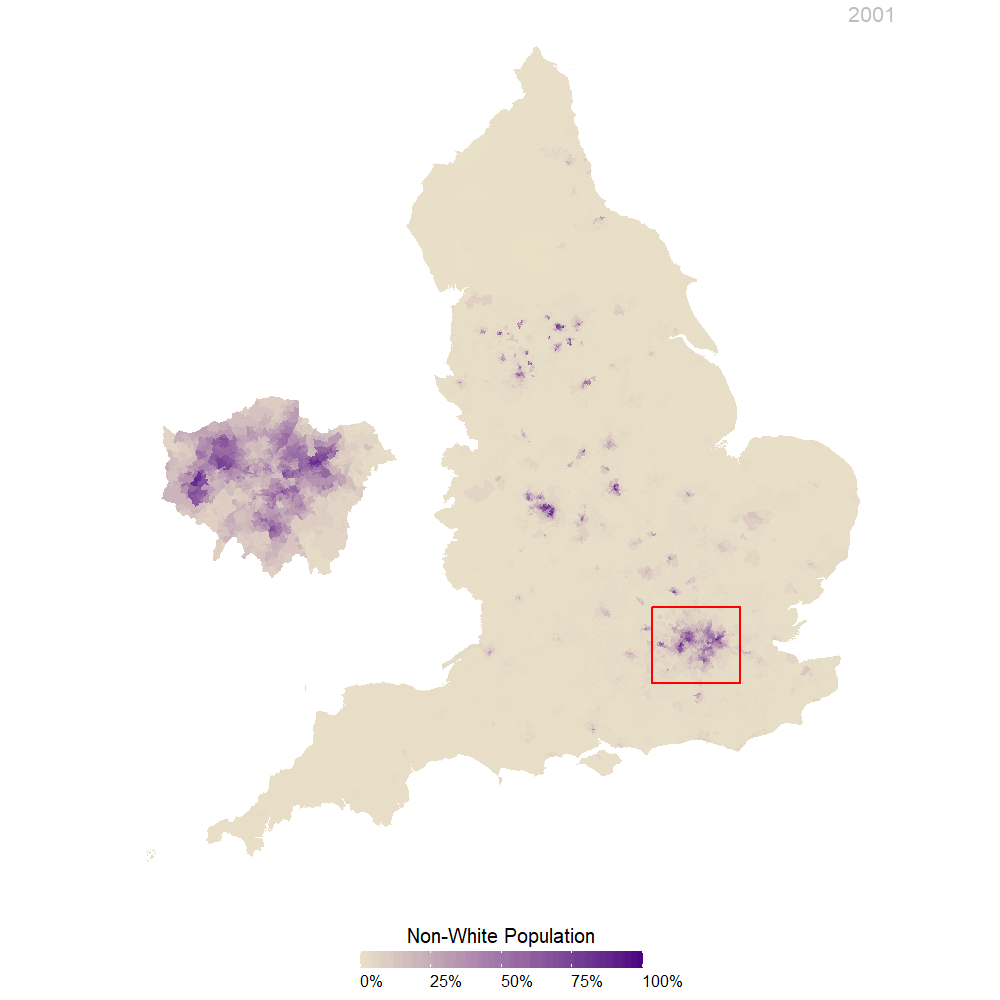

Supplement: MSOA11 DIVERSITY [file mmc2.zip › lanepe_101386_mmc2.gif]

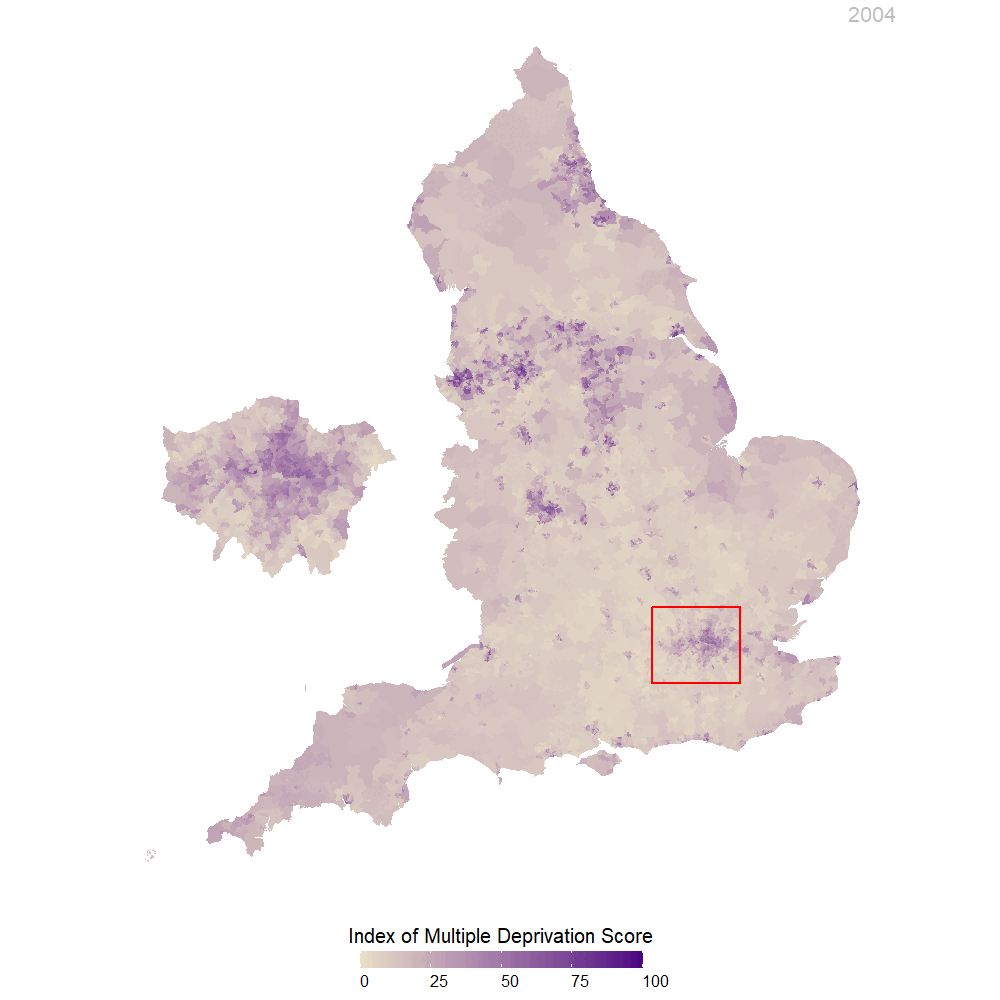

Supplement: MSOA11 IMD [file mmc3.zip › lanepe_101386_mmc3.gif]

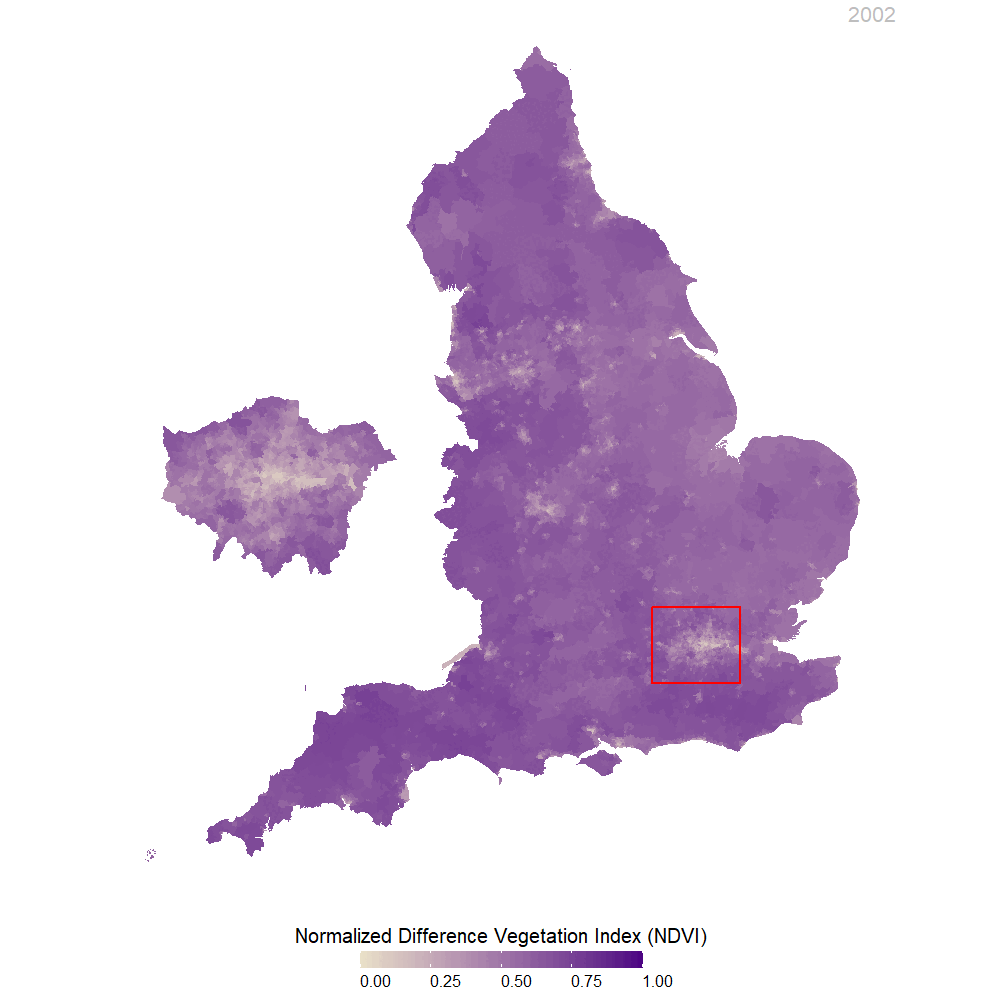

Supplement: MSOA11 NDVI [file mmc4.zip › lanepe_101386_mmc4.gif]

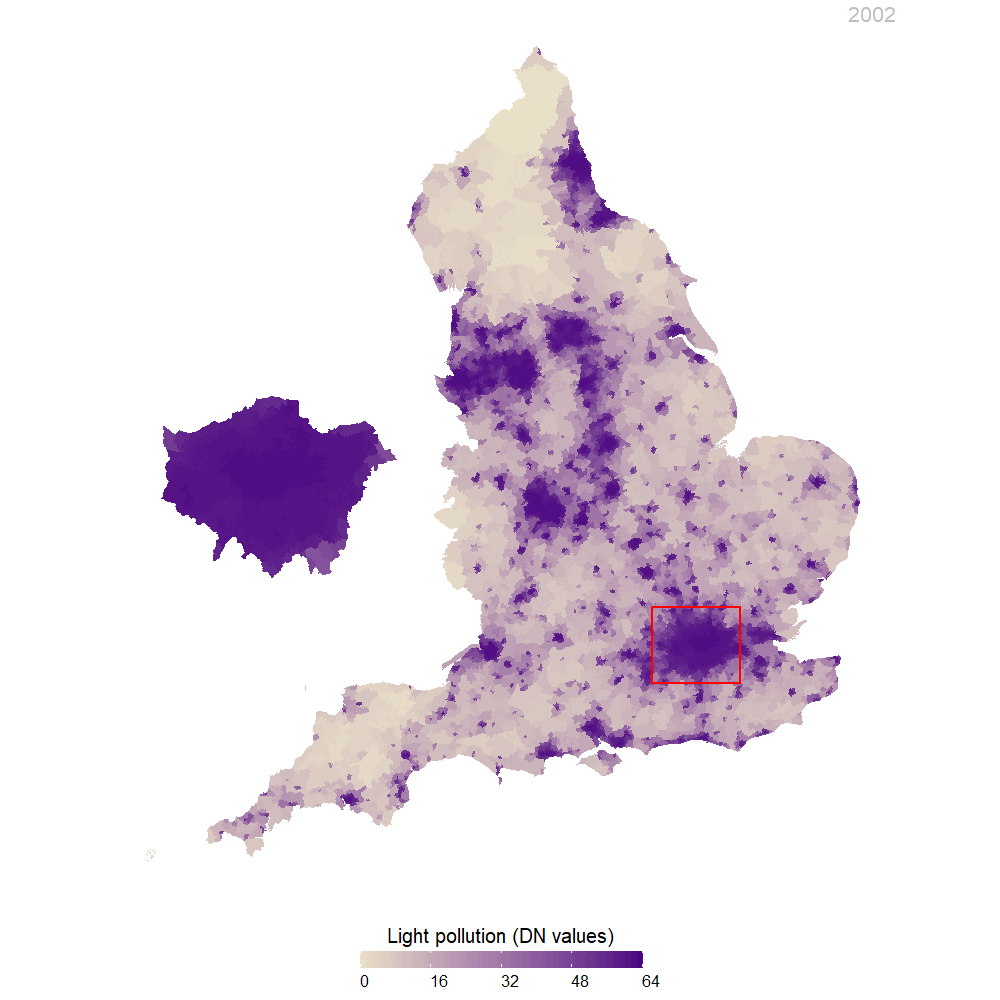

Supplement: MSOA11 NIGHTTIME LIGHT [file mmc5.zip › lanepe_101386_mmc5.gif]

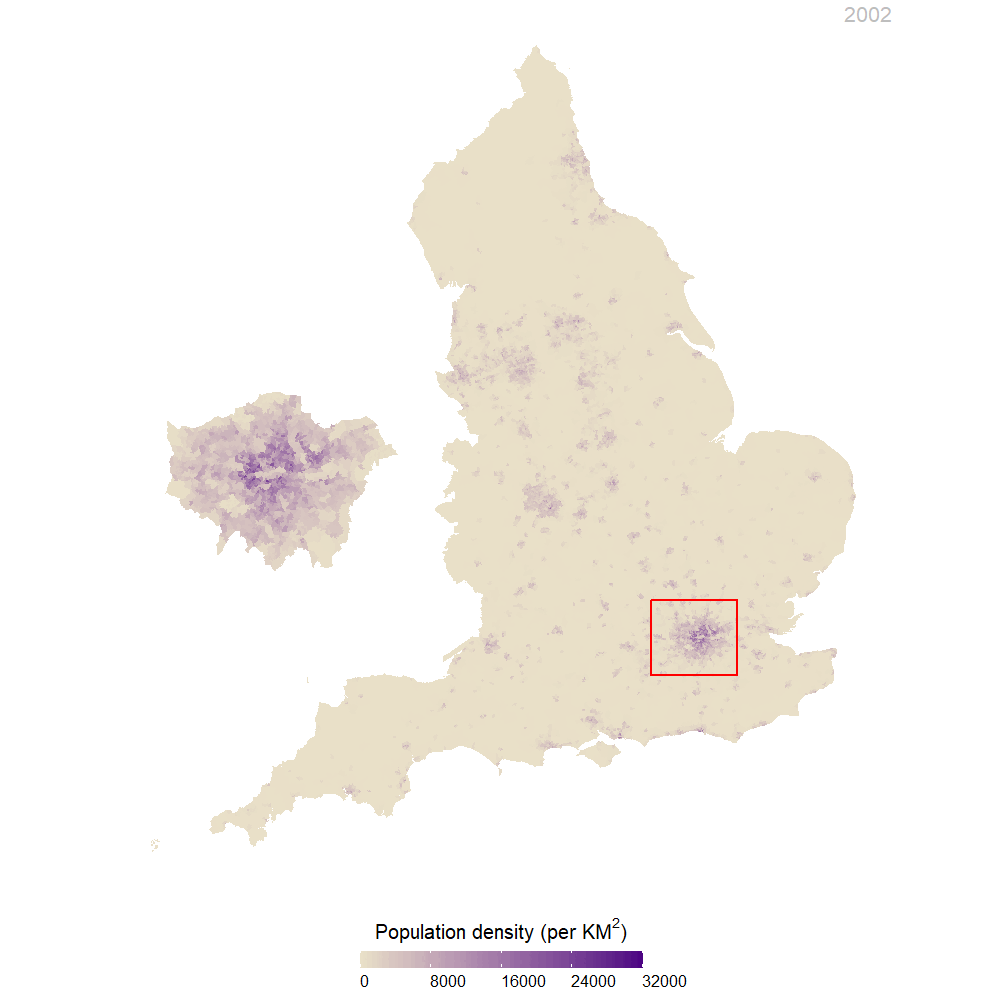

Supplement: MSOA11 POPULATION DENSITY [file mmc6.zip › lanepe_101386_mmc6.gif]

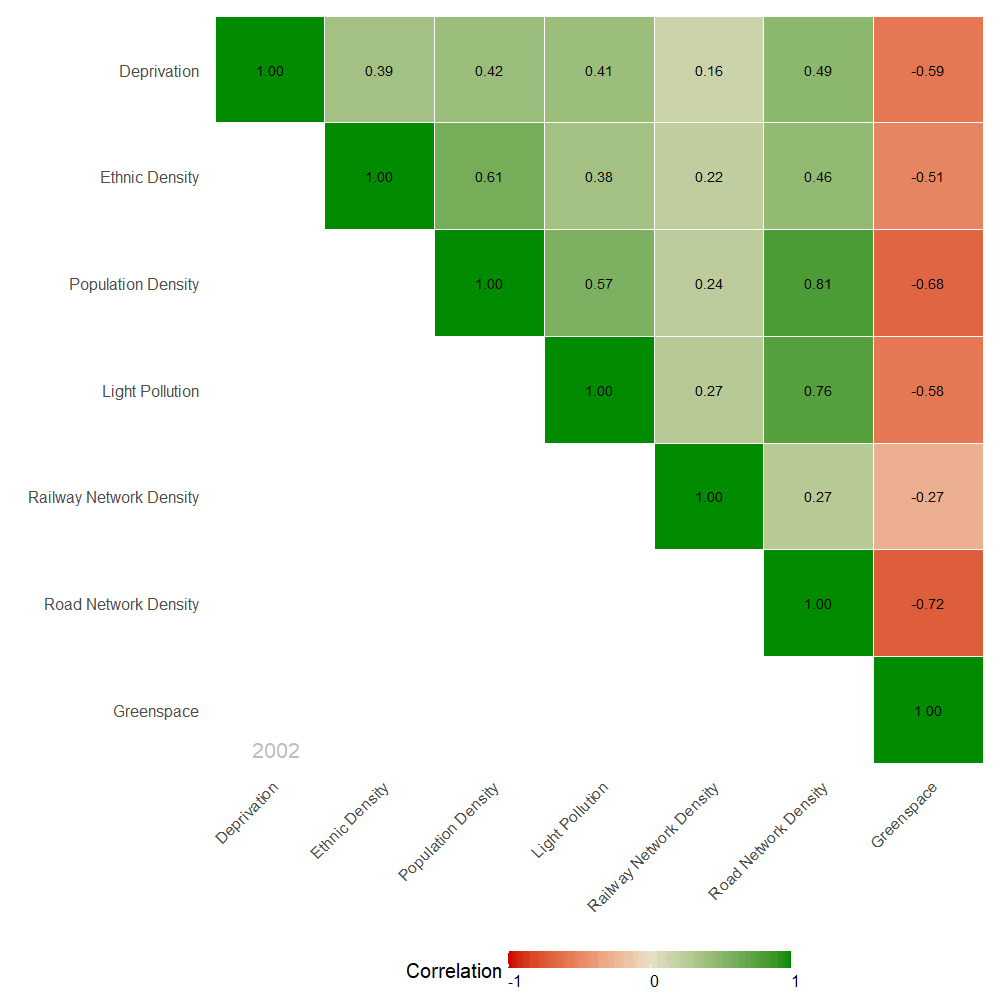

Supplement: CORRELATION MATRIX [file mmc7.zip › lanepe_101386_mmc7.gif]
